# The dynamics and behavior of logarithmic type fuzzy difference equation of order two

**DOI:** 10.1371/journal.pone.0309198

**Published:** 2024-10-18

**Authors:** Muhammad Usman, Abdul Khaliq, Muhammad Azeem, Senesie Swaray, Mohamed Kallel

**Affiliations:** 1 Department of Mathematics, Riphah International University, Lahore, Pakistan; 2 Tree Crops Unit, Sierra Leone Agricultural Research Institute, Freetown, Sierra Leone; 3 Department of Physics, Faculty of Sciences and Arts, Northern Border University, Rafha, Saudi Arabia; Universiti Tun Hussein Onn Malaysia, MALAYSIA

## Abstract

Fuzzy difference equations are becoming increasingly popular in fields like engineering, ecology, and social science. Difference equations find numerous applications in real-life problems. Our study demonstrates that the logarithmic-type fuzzy difference equation of order two possesses a nonnegative solution, and an equilibrium point, and exhibits asymptotic behavior.

$$\begin{array}{c} x_{n+1}=\frac{\alpha +\beta logx_{n}}{A+x_{n-1}},\forall \;n\in W \end{array}$$

Where, *x*_*i*_ represents the sequence of fuzzy numbers, and the parameters *α*, *β*, *A*, along with the initial conditions *x*_−1_ and *x*_0_, are positive fuzzy numbers. The characterization theorem is employed to convert each single logarithmic fuzzy difference equation into a set of two crisp logarithmic difference equations within a fuzzy environment. We evaluated the stability of the equilibrium point of the fuzzy system. Utilizing variational iteration techniques, the method of g-division, inequality skills, and a theory of comparison for logarithmic fuzzy difference equations, we investigated the governing equation dynamics, including its boundedness, existence, and both local and global stability analysis. Additionally, we provided some numerical solutions for the equation describing the system to verify our results.

## 1 Introduction

Fuzzy difference equations (FDEs) are mathematical models used to represent complex systems where the relationships between variables are imprecise or uncertain. The solution to a FDE is a set of fuzzy numbers that describe the system’s behavior over time. The significance of finding a solution to a FDE is that many real-world systems exhibit imprecise or uncertain relationships between their variables. By using fuzzy difference equations to model these systems, we can gain a deeper understanding of their behavior and make more accurate predictions about their future behavior. The purpose of finding a solution to a fuzzy difference equation is to provide a quantitative description of the system being modeled. This can be used to make predictions about future behavior, to identify the factors that are most important in determining the system’s behavior, and to design control strategies that optimize the system’s performance. The methodology for finding a solution to a fuzzy difference equation typically involves identifying the variables that are relevant to the system being modeled, defining fuzzy sets that describe the relationships between these variables, and using a set of fuzzy logic operators to combine these sets into a set of fuzzy rules. The behavior of the system over time can be described using these rules as a FDE. The solution to this equation can be obtained using numerical methods, such as iterative algorithms or fuzzy logic reasoning systems. The validity of the solution can be verified through sensitivity analysis, which involves testing the impact of changes in the input variables on the output variables.

In the first instance, Kandel and Byatt [1] proposed the idea of FDE. As a part of their assessment, Q. Zhang, L. Yang, and D.Liao [2] provided non-zero results of (FDE). Their goal is to demonstrate that the non-zero solution is bounded and persistent. Many recent scholars have been fascinated by the theory and applications of difference equations as it has become increasingly important in applied mathematics to study their behavior and solutions. Agarwal wrote a monograph on difference equations and inequalities in 1992 [3]. This book provides a detailed review of what has been done in the field so far. 1993 was the year Kocic and Ladas [4] proposed the applications on the global behavior of non-linear difference equations of higher order. Researchers have studied the dynamical behavior of non-linear difference equations.

On the role of community structure in evolution of opinion formation [5–7]. Bifurcations, chaotic behavior, and optical solutions for the complex Ginzburg–Landau equation [8–10]. Exact solutions and dynamic properties of a nonlinear fourth-order time-fractional partial differential equation [11, 12].

An investigation of the qualitative behavior of positive solutions of a second-order rational fuzzy difference equation with initial conditions of positive fuzzy numbers and parameters of positive fuzzy numbers is conducted by Q Din [13] i.e given by:

$$\begin{array}{c} x_{n+1}=\frac{x_{n}-x_{n-1}}{P+x_{n}x_{n-1}},\;n=0,1,2.. \end{array}$$

In which P is a +ve FN and *x*_1_, *x*_0_ are +ve FNs.

Q Din, K Khan, and A Nosheen [14] studied the following system of exponential difference equations. They investigated boundedness characteristics, persistence, the existence and uniqueness of positive equilibrium, local and global behavior, and the rate at which positive solutions converge:

$$\begin{array}{c} x_{n+1}=\frac{A_{1}+B_{1}e^{-x_{n}}+C_{1}e^{-x_{n-1}}}{a_{1}+b_{1}y_{n}+c_{1}y_{n-1}},\;y_{n+1}=\frac{A_{2}+B_{2}e^{-y_{n}}+C_{2}e^{-y_{n-1}}}{a_{2}+b_{2}x_{n}+c_{2}x_{n-1}},\;n=0,1,2,.. \end{array}$$

Where the given parameters *A*_*i*_, *B*_*i*_, *C*_*i*_, *a*_*i*_, *b*_*i*_, *c*_*i*_ and initial condition *x*_0_, *x*_−_1, *y*_0_, *y*_−_1 are all +ve real numbers.

The following systems of difference equations were examined by S. Kalabusic, MRS Kulenovic, and E. Pilav [15]:

$$\begin{array}{c} u_{n+1}=\frac{A_{1}+B_{1}u_{n}}{C_{1}+v_{n}},\;v_{n+1}=\frac{A_{2}+B_{2}v_{n}}{C_{2}+u_{n}},\;n=0,1,2... \end{array}$$

where *A*_1_, *B*_1_, *C*_1_, *A*_2_, *B*_2_, and *C*_2_ are +ve numbers, and x0 and y0 are arbitrary non -ve numbers. In this study, the authors demonstrated a rich dynamic of the system that depends on the region of parametric space.

Two systems of second-order rational difference equations were studied by M. N. Qureshi, A. Q. Khan, and Q. Din [16]:

$$\begin{array}{c} x_{n+1}=\frac{ax_{n-1}}{b+cy_{n}^{2}},\;y_{n+1}=\frac{a_{1}y_{n-1}}{b_{1}+c_{1}x_{n}^{2}}\;n=0,1,2,3\ldots \end{array}$$

and

$$\begin{array}{c} x_{n+1}=\frac{dy_{n-1}}{e+fx_{n}^{2}},\;y_{n+1}=\frac{d_{1}x_{n-1}}{e_{1}+f_{1}y_{n}^{2}}\;n=0,1,2,3\ldots \end{array}$$

for all given parameters *a*, *b*, *c*, *a*_1_, *b*_1_, *c*_1_, *d*, *e*, *f*, *d*_1_, *e*_1_, *f*_1_ and initial condition *x*_0_, *x*_1_, *y*_0_, *y*_1_ are all +ve real numbers.

In two systems of exponential difference equations, A.Q. Khan [17] studied the character and persistence of boundedness, the existence and uniqueness of the positive equilibrium, and the rates at which +ve solutions arrive that is given by:

$$\begin{array}{c} x_{n+1}=\frac{Ae^{-y_{n}}+Be^{-y_{n-1}}}{C+A_{1}y_{n}+B_{1}y_{n-1}},\;y_{n+1}=\frac{A_{1}e^{-x_{n}}+B_{1}e^{-x_{n-1}}}{C_{1}+A_{1}x_{n}+B_{1}x_{n-1}},\;n=0,1,2,.. \end{array}$$

for all given parameters *A*, *B*, *C*, *A*_1_, *B*_1_, *C*_1_ and initial condition *x*_0_, *x*_1_, *y*_0_, *y*_1_ are all +ve real numbers.

The following system of difference equations is shown to be asymptotically stable by Ibrahim Yalcinkaya [18]:

$$\begin{array}{c} u_{n+1}=\frac{w_{n}u_{n-1}+b}{w_{n}+u_{n-1}},\;w_{n+1}=\frac{u_{n}w_{n-1}+b}{u_{n}+w_{n-1}},\;n=0,1,2\ldots \end{array}$$

Where parameter *b*, initial condition *u*_0_, *u*_−_1, *w*_0_, and *w*_−_1 be the +ve real numbers.

The periodic nature and form of solutions to the following rational difference equations systems are discussed by N. Touafek and E.M. Elsayed [19]:

$$\begin{array}{c} u_{n+1}=\frac{u_{n-3}}{\pm 1\pm u_{n-3}v_{n-1}},\;v_{n+1}=\frac{v_{n-3}}{\pm 1\pm v_{n-3}u_{n-1}}\;n=0,1,2,3\ldots \end{array}$$

In which we have non zeros initial condition.

Qianhong Zhang, Jingzhong Liu, and Zhenguo Luo [20] investigate the positivity, continuity, and globallay asymptotic stability of solutions for a system of third-order rational difference equations i.e:

$$\begin{array}{c} u_{n+1}=D+\frac{u_{n}}{v_{n-1}v_{n-2}},\;v_{n+1}=D+\frac{v_{n}}{u_{n-1}u_{n-2}}\;n=0,1,2,3\ldots \end{array}$$

Where *D*_*i*_, *u*_−_*i*, and *v*_−_*i* be the all +ve real numbers.

Qianhong Zhang, Lihui Yang, and Jingzhong Liu [21] examined the dynamic characteristics of positive solutions in a third-order rational difference equation system:

$$\begin{array}{c} x_{n+1}=\frac{x_{n-2}}{C+y_{n-2}y_{n-1}y_{n}},\;y_{n+1}=\frac{y_{n-2}}{D+x_{n-2}x_{n-1}x_{n}}\;n=0,1,2,3\ldots \end{array}$$

Where *C*_*i*_, *D*_*i*_, *x*_−_*i*, and *y*_−_*i* be the all +ve real numbers.

According to Deeba et.al [22] the difference equation of degree one was proposed with the historical background of this equation, as presented in this paper.

$$\begin{array}{c} D_{t+1}=a_{1}D_{t}+b_{1},\;t\in W \end{array}$$

It is given that *D*_*t*_, *a*_1_, *b*_1_ belongs to the genetic population and *D*_*t*_, *a*_1_, *b*_1_ belongs to the sequence of fuzzy number that appears in genetic data. As time passed, Deeba and Korvin [22] studied and described linear fuzzy equations of second degree.

$$\begin{array}{c} E_{n+1}=E_{n}-g_{1}h_{1}E_{n-1}+p_{1},\;n=0,1,2,\ldots \end{array}$$

The sequence *E*_*n*_ consists of non-zero (FN) and *g*_1_, *h*_1_, *p*_1_, *E*_0_, *E*_1_ are fuzzy numbers. In the above equation, *CO*_2_ in the human blood is measured using a linearized version of the nonlinear form of a fuzzy difference equation. Two mathematicians [23] in the year 2003 proposed the uniqueness, persistence, existence, and boundedness of non-zero results of similar equations. Through the construction of a Lyapunov-type function and the calculation of ODE, the comparison theorem of the fuzzy difference equation could be obtained. The multiplier method was used by Mondal et al [24] to study a second-order linear (FDE). Hukuhara difference (H-difference) as a tool to calculate (FN), Khastan [25] investigated fuzzy Logistic difference equations and derived global behavior and solution for both equations. According to Papaschinopoulos and Papadopoulos [26], Zadeh Extension Principle can be used to study the global behavior of the following (FDE):

$$\begin{array}{c} u_{n+1}=G+\frac{H}{u_{n}} \end{array}$$

Where G, H are positive (FN). In addition, G. Papaschinopoulos, B.K. Papadopoulos [27] investigated this (FDE):

$$\begin{array}{c} t_{n}+1=A+\frac{t_{n}}{t_{n-m}}. \end{array}$$

Where *A* is a +ve (FN) and *m* = 1, 2. Stefanini [24] finds out a new method by using a general method for dividing (FN) called g-division. A major benefit of g-division is that it reduces imprecision in fuzzy solutions by decreasing the length of the supports. Zhang et al [28] investigated the global behavior of rational (FDE) of order third using g-division of (FN). Also, the same model was later studied by Khastan and Alijani [26]. The solutions obtained by the Zadeh Extension Principle have smaller diameters than the solutions obtained by these researchers. Further, G-division in (FDE) has been the impotent subject of much recent research [29, 30].

In the early years, Zhang et al. [28] studied the existence and the asymptotic behavior of non-zero results of non-linear(FDE).

$$\begin{array}{c} z_{i+1}=\frac{A_{1}z_{i}+Z_{i-1}}{B_{1}+z_{i-1}},\;i=0,1,2,\ldots \end{array}$$

Where *z*_*i*_ is a sequence of non-zero (FN) and *A*_1_, *B*_1_ are non-zero FN where initial conditions *z*_−1_, *z*_0_ are non-zero (FN).

To see discrete-time models in a broader context, could you provide insights into the discrete systems used in the logistic quota harvesting model? Under uncertainty, the difference equation is used to analyze the dynamic behavior of the discrete logistic equation with the Allee effect, provide an overview of discrete models [31–34].

Based on Ozturk et al [35] investigate the second-order crisp type exponential difference equation, the +ve solutions have been studied for their boundedness, convergent rate, and periodicity:

$$\begin{array}{c} x_{i+1}=\frac{\alpha_{1}+\alpha_{2}e^{-x_{i}}}{\alpha_{3}+x_{i-1}},i\in W \end{array}$$

∀ *α*_*i*_ > 0 i.e i = 1,2,3 and *x*_0_, *x*_−1_ be the +ve initial condition.

Q. Din [14] found that +ve equilibrium exists and that it is unique, as well as an understanding of local and global behaviors as a result of this system of exponential type difference equation:

$$\begin{array}{c} u_{i+1}=\frac{A_{1}+B_{1}e^{-u_{i}}+C_{1}e^{-u_{i-1}}}{a_{1}+b_{1}u_{i}+c_{1}u_{i-1}},\;v_{i+1}=\frac{A_{2}+B_{2}e^{-v_{i}}+C_{2}e^{-v_{i-1}}}{a_{2}+b_{2}v_{i}+c_{2}v_{i-1}} \end{array}$$

In which *A*_*i*_, *B*_*i*_, *C*_*i*_, *a*_*i*_, *b*_*i*_, *c*_*i*_∀ (*i* = 1, 2) and initial conditions *u*_0_, *u*_−1_ and *v*_0_, *v*_−1_ are all +ve constants.

In recent decays, Q.Zhange et. al. [36] describe the concept of boundedness, rate of convergence, and characteristics of non-negative solution of an exponential type (FDE) of order two which is given by:

$$\begin{array}{c} z_{i+1}=\frac{A+Be^{-z_{i}}}{C+z_{i-1}},i=0,1,2,\ldots \end{array}$$

Whereas *A*, *B* and *C* be the +ve fuzzy number and *x*_−1_, *x*_0_ be the initial condition.

The motivation behind choosing a model based on fuzzy difference equations is that it allows for the representation of uncertain or imprecise information in a quantitative manner. Fuzzy logic is a mathematical framework that can be used to deal with vague or ambiguous information, and fuzzy difference equations are a specific type of equation that can model dynamic systems with imprecise information. Fuzzy difference equations can be used in various applications, such as control systems, decision-making processes, and prediction models. By incorporating fuzzy logic into the modeling process, the resulting model can better capture the inherent uncertainties and complexities of real-world systems. In addition, fuzzy difference equations can be used to model non-linear and non-deterministic systems, which are often difficult to model using traditional mathematical approaches. This makes them particularly useful in fields such as economics, finance, and engineering, where complex systems are the norm. Overall, the motivation behind choosing a model based on fuzzy difference equations is to improve the accuracy and robustness of modeling by accounting for uncertainty and complexity in the underlying systems. Inspired by above study we explored the dynamic behavior of riccati-type exponential (FDE) of 3rd order.

$$\begin{array}{c} x_{n+1}=\frac{\alpha +\beta \times logx_{n}}{A+x_{n-1}},\;\forall n\in W \end{array}$$ (1)

Where *x*_*n*_ be the sequence of given (FN) and *α*, *β* and *A* be the positive number and *x*_−1_, *x*_0_, be the initial condition. As part of this paper, we aim to determine whether the solution to 1 exists, is unique, and exhibits global behavior using the g-division of (FN) [37]. Problems may be solved in one of two ways, depending on their nature an appropriate formulation may be selected. The modeled behavior of the real world is better described by this model. Our results are an extension of those in [38]. It is not obvious from either result which formulation is best or most similar to the classical case. Some discrete time dynamic models with fuzzy uncertainties can be studied using the method and results. This paper has the following structure. Preliminaries and known results are discussed in Section 2. Following characterization theorems, the existence of an object and its global behavior are guaranteed of (FDE) 1 are obtained in Section 3. Our results are illustrated in Section 4 through two examples. As a final point, Section 5 concludes.

## 2 Preliminaries, definitions and methods

The paper will recall several well-known definitions and results. Please see [37, 39, 40] for more details. All real numbers are designated by *R*(*R*^+^) in this paper.

**Definition 2.1**. [40] *Fuzzy numbers are functions of u*_1_: *R* → [0, 1] *fulfil following conditions*:

*(i) Normality occurs when x*_1_ → *R exists s.t u*(*x*_1_) = 1.

*(ii) u*_1_ *is a fuzzy convex, i.e* ∀ 0 ≤ *t*_1_ ≤ 1, *and* (*x*_1_, *x*_2_) ∈ *R we have u*_1_(*t*_1_*x*_1_ + (1−*t*_1_)*x*_2_) ≥ *min* (*u*_1_(*x*_1_), *u*_2_(*x*_2_)).

*(iii) u*_1_ *is semi continuous to the upper side*.

*(iv) The support of u*_1_, *which is given by* $supp\left(u_{1}\right)=\bar{\cup_{\alpha \in (0,1]}{\left[u_{1}\right]}_{\alpha }}=\left(\bar{x_{1}:u_{1}\left(x_{1}\right)>o}\right)$, *is compact*.

*The α cut of FN u*_1_, *with* 0 ≤ *α* ≤ 1 *is defined by* [*u*_1_]_*α*_ = (*x*_1_ ∈ *R*: *u*_1_(*x*_1_) ≥ *α*). *In particularly, we have* $supp\left(u_{1}\right)={\left[u_{1}\right]}^{0}=\left(\bar{x_{1}\in R|u_{1}\left(x_{1}\right)>0}\right)$.

In the following definition, a (FN) can also be referred to as a pair of functions.

**Definition 2.2**. [40] *It will be helpful to understand how fuzzy numbers are believed to work as a pair of functions* (*v*_*l*_, *v*_*r*_), *with v*_*l*_(*α*), *v*_*r*_(*α*): [0, 1] → *R*, *and the given properties*:

*(i) In mathematical terms*, *v*_*l*_(*α*) *represents a left continuous function with monotonic increasing trend*.

*(ii) v*_*r*_(*α*) *is a monotonically decreasing, continuous function from the left side with bounded value*;

*(iii) v*_*l*_(*α*) ≤ *v*_*r*_(*α*), *α* ∈ [0, 1].

*If a number x*_1_ ∈ *R is reflected by* (*v*_*l*_(*α*), *v*_*r*_(*α*)) = (*x*_1_, *x*_1_), *α* ∈ [0, 1]. *FN have the form of a convex cone that is embedded both the Banach space is isomorphic and isometric* [27] *R*_*F*_ = [*v*|*v* = (*v*_*l*_(*α*), *v*_*r*_(*α*)), 0 ≤ *α* ≤ 1].

Following is a definition of the metric on the space of fuzzy number

**Definition 2.3**. [40] *For u*_1_, *v*_1_ ∈ *R*_*f*_, *then the distance between u*_1_, *and v*_1_ *is given by*:

*D*(*u*_1_, *v*_1_) = sup_*α*∈[0, 1]_ *max*(|*u*_1_*l*, *α* − *v*_1_*l*, *α*|, |*u*_1_*r*, *α* − *v*_1_*r*, *α*|).

*Then its clear that* (*R*_*f*_, *D*) *is a complete metric space*.

**Definition 2.4**. [40] *Let p* = (*p*_*l*_(*α*), *p*_*r*_(*α*)), *q* = (*q*_*l*_(*α*), *q*_*r*_(*α*)) ∈ *R*_*f*_, 0 ≤ *α* ≤ 1, *k*_1_ ∈ *R*. *Then*

*(i) p* = *q* *iff p*_*l*_(*α*) = *q*_*l*_(*α*), *p*_*r*_(*α*) = *q*_*r*_(*α*).

*(ii) p* + *q* = (*p*_*l*_(*α*) + *q*_*l*_(*α*), *p*_*r*_(*α*) + *q*_*r*_(*α*)).

*(iii) pq* = (*p*_*l*_(*α*)*q*_*r*_(*α*), *p*_*r*_(*α*)*q*_*l*_(*α*)).

*(iv)* $wp=\{\begin{array}{c} wp_{l}\left(\alpha \right),wp_{r}\left(\alpha \right)),w\geq 0;\\ (wp_{r}\left(\alpha \right),wp_{l}\left(\alpha \right)),w\leq 0, \end{array}$

*(v) p*.*q* = (*z*_*l*_(*α*), *z*_*r*_(*α*));

*Where*

*z*_*l*_(*α*) = *min*[*p*_*l*_(*α*)*q*_*l*_(*α*), *p*_*l*_(*α*)*q*_*r*_(*α*), *p*_*r*_(*α*)*q*_*l*_(*α*), *p*_*r*_(*α*)*q*_*r*_(*α*)],

*z*_*r*_(*α*) = *max*[*p*_*l*_(*α*)*q*_*l*_(*α*), *p*_*l*_(*α*)*q*_*r*_(*α*), *p*_*r*_(*α*)*q*_*l*_(*α*), *p*_*r*_(*α*)*q*_*r*_(*α*))].

**Definition 2.5**. [39] *A triangular fuzzy number is a triplet D* = (*a*_1_, *b*_1_, *c*_1_) *with the membership function*;

$D\left(x_{1}\right)=\{\begin{array}{c} 0\;\Leftrightarrow \;x_{1}\leq a_{1};\\ \frac{x_{1}-a_{1}}{b_{1}-a_{1}}\;\Leftrightarrow \;a_{1}\leq x_{1}\leq b_{1}\\ 1\;\Leftrightarrow \;x_{1}=b_{1}\\ \frac{c_{1}-x_{1}}{c_{1}-b_{1}}\;\Leftrightarrow \;b_{1}\leq x_{1}\leq c_{1}\\ 0\;iff\;x_{1}\geq \;c_{1} \end{array}$

*The α-cuts of D* = (*a*_1_, *b*_1_, *c*_1_) *are defined by* [*D*]_*α*_ = *x*_1_ ∈ *R*: *D*(*x*_1_) ≥ *α* = [*a*_1_ + *α*(*b*_1_*a*_1_), *c*_1_*α*(*c*_1_*b*_1_)] = [*D*_*l*,*α*_, *D*_*r*_, *α*], *α* ∈ [0, 1]. *Clearly the* [*D*]_*α*_ *are closed intervals*. *If supp D* ⊂ (0, ∞), *then D is said to be a positive FN. It is necessary for us to use the Stacking Theorem given in* [40].

**Theorem 2.1**. *Assume that* (*D*_*α*_: *α* ∈ [0, 1]) *is the family of convex, non-empty and compact subsets of R s.t*:-

*(i)* $\bar{\cup D_{\alpha }}\subset D^{0}$

*(ii)* $D_{2}^{\alpha }\subset D_{1}^{\alpha }\;when\;\alpha_{1}\leq \alpha_{2}$

*(iii)* $D^{\alpha }=\cap_{k\geq 1}D_{k}^{\alpha }\;if\alpha_{k}\;i.e\;\alpha >0$

*Then there exist* $u\in R_{F}^{n}$ *which satisfied* [*u*]_*α*_ = *D*_*α*_, *for any* 0 < *α* ≤ 1 *and* ${\left[u\right]}^{0}=\bar{\cup_{0<\alpha \leq 1}D_{\alpha }}\subset D^{0}$.

*A new concept of division between two FN was recently proposed by Stefanini using an analogy of the gH-difference concept* [37].

**Definition 2.6**. [37] *Let A*_1_, *B*_1_ ∈ *R*_*F*_ *with α cuts*, [*A*_1_]_*α*_ = [*A*_1_*l*, *α*, *A*_1_*r*, *α*], [*B*_1_]_*α*_ = [*B*_1_*l*, *α*, *B*_1_*r*, *α*], *where* 0 ∉ [*B*_1_]_*α*_, *for all* 0 ≤ *α* ≤ 1. *The g-division of A*_1_ *and B*_1_ *is defined by C*_1_ = *A*_1_ ÷_*g*_ *B*_1_ *having α* − *cut*, [*C*_1_]_*α*_ = [*C*_1_*l*, *α*, *C*_1_*r*, *α*], *Whereas* ${\left[C_{1}\right]}_{\alpha -1}=\left[\frac{1}{C_{1}r,\alpha },\frac{1}{C_{1}l,\alpha }\right]$

[*C*_1_]_*α*_ = [*A*_1_]_*α*_ ÷_*g*_[*B*_1_]_*α*_ *iff* $\left\{ \begin{array}{c} {\left[A_{1}\right]}_{\alpha }={\left[B_{1}\right]}_{\alpha }{\left[C_{1}\right]}_{\alpha }\\ or\\ {}[B_{1}{]}_{\alpha }={\left[C_{1}\right]}_{\alpha }{\left[A_{1}\right]}^{\alpha -1} \end{array}\right.$

*if C*_1_ *is a proper FN C*_1_*l*, *α is non-decreasing*, *C*_1_*r*, *α* *is decreasing, and C*_1_*l*, 1 ≤ *C*_1_*r*, 1.

**Remark 1**. *According to* [37], *if* $N=L{÷}_{g}M=N\in R_{F}^{+}$, $\forall \;\left(L,M\right)\;\in R_{F}^{+}$ *exists, i.e. if the FN N is positive, then the following two cases can arise*:.

*CaseI: If* $L_{l,\alpha }M_{r,\alpha }\leq L_{r,\alpha }M_{l},\alpha ,\forall \alpha \in \left[0,1\right],then\;N_{l},\alpha =\frac{L_{l},\alpha }{M_{l},\alpha },N_{r},\alpha =\frac{L_{r},\alpha }{M_{r},\alpha }$

*CaseII: If* $L_{l,\alpha }M_{r,\alpha }\geq L_{r,\alpha }M_{l,\alpha },\forall \alpha \in \left[0,1\right],then\;N_{l},\alpha =\frac{L_{r},\alpha }{M_{r},\alpha },N_{r},\alpha =\frac{L_{l},\alpha }{M_{l},\alpha }$

*According to the following definition, FN see* [41, 42] *are bound and persistent*.

**Definition 2.7**. *Hence, if x*_*n*_ *satisfies Equation* (1.1), *it is considered a +ve fuzzy solutions of* (1.1). *The equilibrium of* (1.1) *will be positive if a +ve FN x satisfies Equation* (1.1).

**Definition 2.8**. *Consider* (*x*_*n*_) *as a sequence of +ve FN and we consider x be in* $R_{F}^{+}$. *In this case, lim*_*n*→∞_*D*(*x*_*n*_, *x*) = 0, *which means that x*_*n*_ → *x as n* → ∞.

**Theorem 2.2**. [43] *(Characterization Theorem)*

“*Let us suppose the FDEs enquiry*

$$\begin{array}{c} {\bar{m}}_{i+1}=\bar{g}\left(x_{n},i\right) \end{array}$$ (2)

*and the initial condition* ${\bar{m}}_{i=0}={\bar{m}}_{0}$

*Where g*: *E*_1_ × Z*_≥0_ → *E*_1_ *such that*

*(1) The parameters of of the function are*:

$$\begin{array}{c} \left[g{\left(\left(m_{i},i\right)\right)}_{\alpha }\right]=\left[\underline{g}\left(\underline{m_{n}}\left(\alpha \right),{\bar{m}}_{n}\left(\alpha \right),n,\alpha \right),\bar{g}\left(\underline{m_{n}}\left(\alpha \right),{\bar{m}}_{n}\left(\alpha \right),n,\alpha \right)\right] \end{array}$$

*(2) The operation i.e (functions)* $\underline{g}\left(\underline{m_{n}}\left(\alpha \right),{\bar{m}}_{n}\left(\alpha \right),n,\alpha \right)$ *and* $\bar{g}\left(\underline{m_{n}}\left(\alpha \right),{\bar{m}}_{n}\left(\alpha \right),n,\alpha \right)$ *be the continuous functions if for any ϵ*_1_ > 0, ∃ *a* Δ_1_ > 0 *such that*

$$\begin{array}{cc} |\underline{g}\left(\underline{m_{n}}\left(\alpha \right),{\bar{m}}_{n}\left(\alpha \right),n\right)-\bar{g}\left(\underline{m_{n1}}\left(\alpha \right),{\bar{m}}_{n1}\left(\alpha \right),n_{1}\right)|<\epsilon_{1}\forall & \alpha \in [0,1] \end{array}$$

*with*

$$\begin{array}{cc} \left|\right|\left(\underline{m_{n}}\left(\alpha \right),{\bar{m}}_{n}\left(\alpha \right),n\right)-\left(\underline{m_{n1}}\left(\alpha \right),{\bar{m}}_{n1}\left(\alpha \right),n_{1}\right)|<\Delta_{1}\forall & \alpha \in [0,1] \end{array}$$

*and ϵ*_2_ > 0 *there exist a* Δ_2_ > 0 *such that*

$$\begin{array}{cc} |\underline{g}\left(\underline{m_{n}}\left(\alpha \right),{\bar{m}}_{n}\left(\alpha \right),n,\alpha \right)-\bar{g}\left(\underline{m_{n2}}\left(\alpha \right),{\bar{m}}_{n2}\left(\alpha \right),n_{2}\right)|<\epsilon_{2}\forall & \alpha \in [0,1] \end{array}$$

*with*

$$\begin{array}{cc} \left|\right|\left(\underline{m_{n}}\left(\alpha \right),{\bar{m}}_{n}\left(\alpha \right),n\right)-\left(\underline{m_{n2}}\left(\alpha \right),{\bar{m}}_{n2}\left(\alpha \right),n_{2}\right)|<\Delta_{2}\forall & \alpha \in [0,1] \end{array}$$

*Then the D.E* (1) *reduce the system of 2 D.E as*

$$\begin{array}{c} {\underline{m}}_{n+1}\left(\alpha \right)=\underline{g}\left(\underline{m_{n}}\left(\alpha \right),{\bar{m}}_{n}\left(\alpha \right),n,\alpha \right)\\ {\bar{m}}_{n+1}\left(\alpha \right)=\bar{g}\left(\underline{m_{n}}\left(\alpha \right),{\bar{m}}_{n}\left(\alpha \right),n,\alpha \right) \end{array}$$

*with initial condition*

$$\begin{array}{c} {\underline{m}}_{n=0}\left(\alpha \right)={\underline{m}}_{0}\left(\alpha \right)\\ {\bar{m}}_{n=0}\left(\alpha \right)={\bar{m}}_{0}\left(\alpha \right)” \end{array}$$

**Note 2.1**. Now by applying characterization results of the theorems a single DE is converted into the systems of the 2 crisp DE. In the paper which we study now in the environment of fuzziness theory we take a single DE. And hence, the given DE is converted to 2 crisp DE.

## 3 Main results

### 3.1 Uniqueness and existence of system

To find out and checking the uniqueness and existence of +ve results we have to need the lemma’s which are given blows:

**Lemma 3.1**. [40] *Assume that h be the continuous operation, from G*^+^ × *G*^+^ × *G*^+^ → *G*^+^ *and the given α*_1_, *β*_1_ *and A*_1_ *be the FN. Then*,

$$\begin{array}{c} {\left[h\left(\alpha_{1},\beta_{1},A_{1}\right)\right]}_{\alpha }=h\left({\left[\alpha_{1}\right]}_{\alpha },{\left[\beta_{1}\right]}_{\alpha },{\left[A_{1}\right]}_{\alpha }\right),\;0\;\leq \alpha \;\leq 1 \end{array}$$ (3)

**Theorem 3.1**. *Assume the* Eq 1, *in which x*_*n*_ *be the sequence of (FN) and α*, *β*, *A be the non-zero constant x*_−1_, *x*_0_ *are the initial conditions. There exist a unique non-zero solutions x*_*n*_ *of equation (1.1) with initial conditions x*_−1_, *x*_0_.

*Proof*. The proof is same as one of the Theorem 3.1 in [22]. Suppose there exist, a sequence of FN *x*_*n*_ which hold the equations (1) with initial condition *x*_−1_, *x*_0_. Now consider the *α* − *levelcut*, 0 ≤ *α* ≤ 1,

$$\begin{array}{c} {\left[\alpha \right]}_{\alpha }=\left[\alpha_{l,\alpha },\alpha_{r,\alpha }\right]\;and\;\;{\left[\beta \right]}_{\alpha }=\left[\beta_{l,\alpha },\beta_{r,\alpha }\right]\;and\;\;{\left[A\right]}_{\alpha }=\left[A_{l,\alpha },A_{r,\alpha }\right],\\ \;n=-1,0\ldots , \end{array}$$ (4)

Lemma (3.1), it follows that

$$\begin{array}{c} {\left[x_{n+1}\right]}_{\alpha }=\left[L_{n+1,\alpha }^{\prime },R_{n+1,\alpha }^{\prime }\right]=[\frac{\alpha +\beta logx_{n}}{A+x_{n-1}}{]}_{\alpha }=\frac{{\left[\alpha \right]}_{\alpha }+{\left[\beta \right]}_{\alpha }\times {\left[logx_{n}\right]}_{\alpha }}{{\left[A\right]}_{\alpha }+{\left[x_{n-1}\right]}_{\alpha }}\\ =\frac{\left[\alpha_{l,\alpha }+\beta_{l,\alpha }\times logR_{n,\alpha }^{\prime },\alpha_{r,\alpha }+\beta_{r,\alpha }\times logL_{n,\alpha }^{\prime }\right]}{\left[A_{l},\alpha +L_{n-1}^{\prime },\alpha ,A_{r}\alpha +R_{n-1,\alpha }^{\prime }\right]} \end{array}$$

According to Remark (1), we have two cases for discussion which is given by:

Case I:

$$\begin{array}{c} {\left[x_{n+1}\right]}_{\alpha }=\left[L_{n+1,\alpha },R_{n+1,\alpha }\right]=[\frac{\alpha_{l,\alpha }+\beta_{l,\alpha }\times logR_{n,\alpha }}{A_{l,\alpha }+L_{n-1,\alpha }},\frac{\alpha_{r,\alpha }+\beta_{r,\alpha }\times logL_{n,\alpha }}{A_{r,\alpha }+R_{n-1,\alpha }}] \end{array}$$ (5)

Case II:

$$\begin{array}{c} {\left[x_{n+1}\right]}_{\alpha }=\left[L_{n+1,\alpha }^{\prime },R_{n+1,\alpha }^{\prime }\right]=[\frac{\alpha_{r,\alpha }+\beta_{r,\alpha }\times logL_{n,\alpha }^{\prime }}{A_{r,\alpha }+R_{n-1,\alpha }^{\prime }},\frac{\alpha_{l,\alpha }+\beta_{l,\alpha }\times logR_{n,\alpha }^{\prime }}{A_{l,\alpha }+L_{n-1,\alpha }^{\prime }}] \end{array}$$ (6)

When Case I becomes true then

$$\begin{array}{c} \frac{\alpha_{l,\alpha }+\beta_{l,\alpha }*logR_{n,\alpha }^{\prime }}{\alpha_{r,\alpha }+\beta_{r,\alpha }\times logL_{n,\alpha }^{\prime }}\leq \frac{A_{l,\alpha }+L_{n-1,\alpha }^{\prime }}{A_{r,\alpha }+R_{n-1,\alpha }^{\prime }} \end{array}$$

for all *n* ≥ 0 and 0 < *α* ≤ 1, Now

$$\begin{array}{cc} \;L_{n+1,\alpha }^{\prime } & =\frac{\alpha_{l,\alpha }+\beta_{l,\alpha }\times logR_{n,\alpha }^{\prime }}{A_{l,\alpha }+L_{n-1,\alpha }^{\prime }}\\ \;R_{n+1,\alpha }^{\prime } & =\frac{\alpha_{r,\alpha }+\beta_{r,\alpha }\times logL_{n,\alpha }^{\prime }}{A_{r,\alpha }+R_{n-1,\alpha }^{\prime }} \end{array}$$ (7)

It is clear that initial conditions $\left[L_{n,\alpha }^{\prime },R_{n,\alpha }^{\prime }\right]$. (*n* = −1, 0) and *α* ∈ (0, 1] then the system 7 has a unique solution of $\left(L_{n,\alpha }^{\prime },R_{n,\alpha }^{\prime }\right)$. Alternately, we want to show that $[L_{n+1,\alpha }^{\prime },R_{n+1,\alpha }^{\prime }]\;0\leq \alpha \leq 1$ where $L_{n,\alpha }^{\prime },R_{n,\alpha }^{\prime }$ is the results of the systems 7 *n* = −1, 0

$${\left[x_{n}\right]}_{\alpha }=[L_{n,\alpha }^{\prime },R_{n,\alpha }^{\prime }]\;,0\leq \alpha \leq 1,\forall n\;\in W$$ (8)

Put *n* = 1 and since *α*, *β*, *A* and *x*_*n*_*bethenonnegative*(*FN*)*forall*(*n* = −1, 0..). It is simple to see that $\left[L_{l,\alpha }^{\prime },R_{l,\alpha }^{\prime }\right]$ is the *α*-cut of $x_{1}=\frac{\alpha +\beta \times logx_{1}}{A+x_{-1}}$, for any *α* ∈ (0, 1]. Now we have

$$\begin{array}{c} \left[L_{1,\alpha }^{\prime },R_{1,\alpha }^{\prime }\right]=[\frac{\alpha_{l,\alpha }+\beta_{l,\alpha }\times logR_{1,\alpha }^{\prime }}{A_{l,\alpha }+L_{-1,\alpha }^{\prime }},\frac{\alpha_{r,\alpha }+\beta_{r,\alpha }\times logL_{1,\alpha }^{\prime }}{A_{r,\alpha }+R_{-1,\alpha }^{\prime }}]=\frac{{\left[\alpha \right]}_{\alpha }+{\left[\beta \right]}_{\alpha }\times {\left[logx_{1}\right]}_{\alpha }}{{\left[A\right]}_{\alpha }+{\left[x_{-1}\right]}_{\alpha }} \end{array}$$

By working on it inductively, now let $\left[L_{k}^{\prime },\alpha ,R_{k}^{\prime },\alpha \right]$ is the required *α* -cuts of *x*_*k*_ i.e ${\left[x_{k}\right]}_{\alpha }=\left[L_{k}^{\prime },\alpha ,R_{k}^{\prime },\alpha \right]$. Now we have to show that $\left[l_{k+1},\alpha ,R_{k+1}^{\prime },\alpha \right]$ has the *α* -level of

$$\begin{array}{c} x_{k+1}=\frac{\alpha +\beta *logx_{k}}{A+x_{k-1}}. \end{array}$$

According to (7) for any *α* ∈ (0, 1] we have

$$\begin{array}{c} \left[L_{k+1,\alpha }^{\prime },R_{k+1,\alpha }^{\prime }\right]=[\frac{\alpha_{l,\alpha }+\beta_{l,\alpha }\times logR_{k,\alpha }^{\prime }}{A_{l,\alpha }+L_{k-1,\alpha }^{\prime }},\frac{\alpha_{r,\alpha }+\beta_{r,\alpha }\times logL_{k,\alpha }^{\prime }}{A_{r,\alpha }+R_{k-1,\alpha }^{\prime }}]=\frac{{\left[\alpha \right]}_{\alpha }+{\left[\beta \right]}_{\alpha }\times {\left[logx_{k}\right]}_{\alpha }}{{\left[A\right]}_{\alpha }+{\left[x_{k-1}\right]}_{\alpha }} \end{array}$$

$$\begin{array}{c} =[\frac{\alpha +\beta logx_{k}}{A+x_{k-1}}{]}_{\alpha } \end{array}$$ (9)

As $\left[L_{k+1,\alpha }^{\prime },R_{k+1,\alpha }^{\prime }\right]$ is the required *α*-level of the FN $x_{k+1}=\frac{\alpha +\beta \times logx_{k}}{A+x_{k-1}}$. Then for-all n and 0 ≤ *α* ≤ 1, $\left[L_{n,\alpha }^{\prime },R_{n,\alpha }^{\prime }\right]$ is the desired *α*-level of the FN *x*_*n*_

Now we have to show the uniqueness of the required solution. If the 1 has any other results of the type of $\bar{x_{n}}$ with the initial condition *x*_*n*_, *n* = −1, 0. Then we have to use similar arguments as above, i.e

$$\begin{array}{c} \left[\bar{x_{n}},\alpha \right]=\left[L_{n}^{\prime },\alpha ,R_{n}^{\prime },\alpha \right] \end{array}$$ (10)

for all 0 ≤ *α* ≤ 1 and ∀ *n* = 0, 1, 2… Now it is clear from 8 and 10 $\left(x_{n},\alpha \right)=\left(\bar{x_{n}},\alpha \right)$, 0 < *α* ≤ 1, ∀ *n* ∈ *W*.

If case-II is hold, then the proof follows the same argument as case-I so we neglect it. This concluded the proof, to find the qualitative characteristics of third-order exponential types (FDE) 1, we needed to discuss about qualitative behaviour of the given systems for crisp difference equation. According to the g-division we discuss two cases which mention already.

When case-I is hold then the given definitions and two lemmas are necessary to proof the next theorems.

**Definition 3.1**. [4] *Consider, the systems of DE*

$$\begin{array}{c} y_{n+1}=\frac{\alpha_{1}+\beta_{1}\times logz_{n}}{a_{1}+y_{n-1}},\;z_{n+1}=\frac{\alpha_{2}+\beta_{2}\times logy_{n}}{a_{1}+z_{n-1}},\;n\in W \end{array}$$ (11)

*Where α*_*i*_, *β*_*i*_(*α*_*i*_ ≥ *β*_*i*_), *a*_*i*_ (*i* = 0, 1) *and initial condition y*_*i*_, *z*_*i*_, *i* = [−1, 0] *be the +ve real numbers. We have to find a positive-solution of* [*y*_*n*_, *z*_*n*_] *which is bounded and persistence if* ∃ *some +ve constant K and L such that*:

$$\begin{array}{c} K\leq y_{n}\leq L\;and\;K\leq z_{n}\leq L.\;\forall \;n=-1,0\ldots \end{array}$$

**Lemma 3.2**. *Suppose the systems of DE written as above* 11 *Where α*_*i*_, *β*_*i*_, (*α*_*i*_ ≥ *β*_*i*_), *a*_*i*_ *i* = [1, 2] *and initial condition y*_*i*_, *z*_*i*_, *i* = (−1, 0) *be the positive number. Then the two condition hold*:

(*i*) *The +ve results of* 11 *is exist which is bounded*.

(*ii*) *The systems admits a unique +ve EP i.e* $\left(\bar{y},\bar{z}\right)$ ∈ (0, *L*_1_] × (0, *L*_2_] *which are the locally, asymptotically stable where*:

$$\begin{array}{c} L_{i}=\frac{\alpha_{i}+\beta_{i}}{a_{i}},i=1,2\;\;if\;\;log\frac{a_{1}}{2}\geq \frac{\beta_{1}\beta_{2}}{a_{1}a_{2}}, \end{array}$$ (12)

*and then*

$$\begin{array}{c} \frac{\alpha_{1}+\beta_{1}}{{a_{1}}^{2}}+\frac{\alpha_{1}+\beta_{1}}{{a_{1}}^{2}}.\frac{\alpha_{2}+\beta_{2}}{{a_{2}}^{2}}-\frac{\alpha_{2}+\beta_{2}}{{a_{2}}^{2}}<1 \end{array}$$ (13)

*Proof*. For (i) let (*y*_*n*_, *z*_*n*_) be the any +ve results of 11 then,

$$\begin{array}{c} y_{n}\leq \frac{\alpha_{1}+\beta_{1}}{a_{1}}=L_{1},z_{n}\leq \frac{\alpha_{2}+\beta_{2}}{a_{2}}=L_{2} \end{array}$$ (14)

Now from 11 and 14 we have

$$\begin{array}{c} y_{n}\geq \frac{\alpha_{1}+\beta_{1}log\frac{\alpha_{2}+\beta_{2}}{a_{2}}}{a_{1}+\frac{\alpha_{1}+\beta_{1}}{a_{1}}}=K_{1},\;z_{n}\geq \frac{\alpha_{2}+\beta_{2}log\frac{\alpha_{1}+\beta_{1}}{a_{1}}}{a_{2}+\frac{\alpha_{2}+\beta_{2}}{a_{2}}}=K_{2} \end{array}$$ (15)

From above we have,

$$\begin{array}{c} K_{1}\leq y_{n}\leq L_{1},\;\;K_{2}\leq z_{n}\leq L_{2} \end{array}$$

Which shows that the +ve results of 11 is bounded and existence.

(ii) Now let the systems

$$\begin{array}{c} y=\frac{\alpha_{1}+\beta_{1}logz}{a_{1}+y},z=\frac{\alpha_{2}+\beta_{2}logy}{a_{2}+z} \end{array}$$ (16)

Now we can write the system 16 in the form of

$$\begin{array}{c} y^{2}+a_{1}y-\alpha_{1}-\beta_{1}logz=0andz^{2}+a_{2}y-\alpha_{2}-\beta_{2}logy=0 \end{array}$$ (17)

From the required system (17), we have

$$\begin{array}{c} y=h\left(z\right)=\frac{-a_{1}+\sqrt{a_{1}^{2}+4\alpha_{1}+4\beta_{1}logz}}{2} \end{array}$$

and also we denoting this as:

$$\begin{array}{c} F\left(z\right)=z^{2}+a_{2}z-\alpha_{2}-\beta_{2}logh(z) \end{array}$$ (18)

Now we obtain *F*(*z*) < 0

and

$$\begin{array}{c} F\left(L_{2}\right)={\left[\frac{\alpha_{2}+\beta_{2}}{a_{2}}\right]}^{2}+a_{2}\frac{\alpha_{2}+\beta_{2}}{a_{2}}-\alpha_{2}-\beta_{2}logh(L_{2})>0 \end{array}$$

Therefore, we observe that there exist at least one positive solution *z* ∈ (0, *L*_2_]

From the Eqs 12 and 18, we have

$$F^{\prime }\left(z\right)=2z+a_{2}+\beta_{2}logh(z)h^{\prime }\left(z\right)=2z+a_{2}-\frac{\beta_{2}}{-a_{1}+\sqrt{a_{1}^{2}+4\alpha_{1}+4\beta_{1}logz}}\times \frac{2\beta_{1}\frac{1}{z}}{\sqrt{a_{1}^{2}+4\alpha_{1}+4\beta_{1}logz}}$$

$$\begin{array}{c} \geq a_{1}-2e^{\frac{\beta_{1}\beta_{2}}{a_{1}a_{2}}}>0. \end{array}$$ (19)

Therefore there exist F(z)=0 have a unique +ve EP $\bar{z}\in \left(0,L_{2}\right]$. And also we have a unique +ve EP $\bar{y}\in \left(0,L_{1}\right]$.

Furthermore the J-matrix $J_{\bar{y},\bar{z}}$ of 11 at $\left(\bar{y},\bar{z}\right)$ is given by:

$$\begin{array}{c} J_{\bar{y},\bar{z}}=[\begin{array}{cccc} 0 & D_{1} & E_{1} & 0\\ 1 & 0 & 0 & 0\\ E_{2} & 0 & 0 & D_{2}\\ 0 & 0 & 1 & 0 \end{array}] \end{array}$$

Where $D_{1}=-\frac{\alpha_{1}+\beta_{1}logv}{{\left(a_{1}+u\right)}^{2}}$, $E_{1}=\frac{\beta_{1}}{v\times (a_{1}+u)}$, $E_{2}=\frac{\beta_{2}}{u\times (a_{2}+v)}$, $D_{2}=-\frac{\alpha_{2}+\beta_{2}logu}{{\left(a_{2}+v\right)}^{2}}$,

Now we find the characteristics equation the given $J_{\left(\bar{y},\bar{z}\right)}$ at $\left(\bar{y},\bar{z}\right)$ which is given by:

$$\begin{array}{c} \lambda^{4}-\lambda^{2}\left(D_{1}+D_{2}+E_{1}E_{2}\right)+D_{1}D_{2}=0 \end{array}$$ (20)

Now taking the absolute values of *λ*^3^ and content of these characteristics equation we have:

$$\begin{array}{c} |D_{1}|+D_{1}D_{2}+D_{2}=\left|\frac{\alpha_{1}+\beta_{1}e^{-v}}{{\left(a_{1}+u\right)}^{2}}\right|+\frac{\alpha_{1}+\beta_{1}e^{-v}}{{\left(a_{1}+u\right)}^{2}}.\frac{\alpha_{2}+\beta_{2}e^{-u}}{{\left(a_{2}+v\right)}^{2}}-\frac{\alpha_{2}+\beta_{2}e^{-u}}{{\left(a_{2}+v\right)}^{2}}\leq \\ \frac{\alpha_{1}+\beta_{1}}{{a_{1}}^{2}}+\frac{\alpha_{1}+\beta_{1}}{{a_{1}}^{2}}.\frac{\alpha_{2}+\beta_{2}}{{a_{2}}^{2}}-\frac{\alpha_{2}+\beta_{2}}{{a_{2}}^{2}}<1 \end{array}$$

By the Remarks 1.3.1 of [4], We deduct the equilibrium pints($\bar{y},\bar{z}$) is stable asymptotically.

**Lemma 3.3**. *let the EP* $\left(\bar{y},\bar{z}\right)=\left(\bar{\theta },\bar{\phi }\right)$ *of* 11 *is said to be globally asymptotically stable if*:

$$\begin{array}{c} \alpha_{1}+\beta_{1}logK_{2}<\bar{\theta }\left(a_{1}+K_{1}\right),\alpha_{2}+\beta_{2}logK_{1}<\bar{\phi }\left(a_{2}+K_{2}\right) \end{array}$$ (21)

*Proof*. $\xi_{s}=\bar{\theta }\left(-1+\frac{\theta_{s}}{\bar{\theta }}-\text{ln}\;\frac{\theta_{s}}{\bar{\theta }}\right)+\bar{\phi }\left(-1+\frac{\phi_{s}}{\bar{\phi }}-\text{ln}\;\frac{\phi_{s}}{\bar{\phi }}\right)$.

Since: −1 + *θ* − ln *θ* ≥ 0, *for all θ* > 0, then we have *ξ*_*s*_ ≥ 0. More-over we conclude

$$\begin{array}{c} -\text{ln}\frac{\theta_{s+1}}{\theta_{s}}=\text{ln}\frac{\theta_{s}}{\theta_{s+1}}=\text{ln}\left(1-\left(1-\frac{\theta_{s}}{\theta_{s+1}}\right)\right)\leq -\left(1-\frac{\theta_{s}}{\theta_{s+1}}\right)\leq -\frac{\theta_{s+1}-\theta_{s}}{\theta_{s+1}} \end{array}$$ (22)

$$\begin{array}{c} -\text{ln}\frac{\phi_{s+1}}{\phi_{s}}=\text{ln}\frac{\phi_{s}}{\phi_{s+1}}=\text{ln}\left(1-\left(1-\frac{\phi_{s}}{\phi_{s+1}}\right)\right)\leq -\left(1-\frac{\phi_{s}}{\phi_{s+1}}\right)\leq -\frac{\phi_{s+1}-\phi_{s}}{\phi_{s+1}} \end{array}$$ (23)

Now we take difference of *ξ*_*s*+1_ − *ξ*_*s*_ i.e

$$\begin{array}{c} \xi_{s+1}-\xi_{s}=\bar{\theta }\left(-1+\frac{\theta_{s+1}}{\bar{\theta }}-\text{ln}\frac{\theta_{s+1}}{\bar{\theta }}\right)+\bar{\phi }\left(-1+\frac{\phi_{s+1}}{\bar{\phi }}-\text{ln}\frac{\phi_{s+1}}{\bar{\phi }}\right)-\bar{\theta }\left(-1+\frac{\theta_{s}}{\bar{\theta }}-\text{ln}\frac{\theta_{s}}{\bar{\theta }}\right)+\\ \bar{\phi }\left(-1+\frac{\phi_{s}}{\bar{\phi }}-\text{ln}\frac{\phi_{s}}{\bar{\phi }}\right). \end{array}$$

$$\begin{array}{c} =\left(\theta_{s+1}-\theta_{s}\right)+\left(\phi_{s+1}-\phi_{s}\right)-\bar{\theta }\frac{\theta_{s+1}}{\bar{\theta }}-\bar{\phi }\frac{\phi_{s+1}}{\bar{z}}\leq \left(\theta_{s+1}-\theta_{s}\right)+\left(\theta_{s+1}-\phi_{s}\right)-\bar{\theta }\frac{\theta_{s+1}-\theta_{s}}{\theta_{s+1}}-\bar{\phi }\frac{\phi_{s+1}-\phi_{s}}{\phi_{s+1}}\\ =\left(\theta_{s+1}-\theta_{s}\right)\left(1-\frac{\bar{\theta }}{\theta_{s+1}}\right)+\left(\phi_{s+1}-\phi_{s}\right)\left(1-\frac{\bar{\phi }}{\phi_{s+1}}\right) \end{array}$$

$$\begin{array}{c} =\left(\theta_{s+1}-\theta_{s}\right)\left(1-\frac{\bar{\theta }\left(a_{1}+\theta_{s-1}\right)}{\alpha_{1}+\beta_{1}log\phi_{s}}\right)+\left(\phi_{s+1}-\phi_{s}\right)\left(1-\frac{\bar{\phi }\left(a_{2}+\phi_{s-1}\right)}{\alpha_{2}+\beta_{2}log\theta_{s}}\right) \end{array}$$ (24)

Under condition 21, for any *n* ≥ 0, we have

$$\begin{array}{c} \xi_{s+1}-\xi_{s}\leq \left(L_{1}-K_{1}\right)\left(\frac{\alpha_{1}+\beta_{1}\times logK_{2}-\bar{\theta }\left(a_{1}+K_{1}\right)}{\alpha_{1}+\beta_{1}logK_{2}}\right)\leq 0. \end{array}$$

This implies that lim_*s*→∞_ *ξ*_*s*_ ≥ 0. And also lim_*s*→∞_(*ξ*_*s*−1_ − *ξ*_*s*_) = 0. And thus we conclude that ${\text{lim}}_{s\to \infty }\left(\theta_{s},\phi_{s}\right)=\left(\bar{\theta },\bar{\phi }\right)$.

Now by using condition (ii) of lemma (3.2) we prove that $\left(\bar{\theta },\bar{\phi }\right)$ is globally stable.

**Theorem 3.2**. *Considered the FDE of* (1)

$$\begin{array}{c} \frac{\alpha_{l,\alpha }+\beta_{l,\alpha }\times logR_{n,\alpha }}{\alpha_{r,\alpha }+\beta_{r,\alpha }\times logL_{n,\alpha }}\leq \frac{A_{l,\alpha }+L_{n-1,\alpha }}{A_{r,\alpha }+R_{n-1,\alpha }},\forall \;n\in W\;and\;\alpha \in \left(0,1\right] \end{array}$$ (25)

*Then the two condition hold*:

*(i) Every +ve results x*_*n*_ of (1) *is exist and bounded*.

*(ii) Every +ve results of x*_*n*_ *of* 1 *is converges to only one EP x as n* → ∞ *and* ∀ *α* ∈ (0, 1].

$$\begin{array}{c} A_{l,\alpha }A_{r,\alpha }e^{-\frac{A_{l,\alpha }}{2}}>\beta_{l,\alpha }\beta_{r,\alpha } \end{array}$$ (26)

*And*

$$\begin{array}{c} \frac{\alpha_{l,\alpha }+\beta_{l,\alpha }}{A_{l,\alpha }^{2}}+\frac{\alpha_{r,\alpha }+\beta_{r,\alpha }}{A_{r,\alpha }^{2}}+\frac{\beta_{l,\alpha }.\beta_{r,\alpha }}{A_{l,\alpha }.A_{r,\alpha }}+\frac{\alpha_{l,\alpha }+\beta_{l,\alpha }}{A_{l,\alpha }}.\frac{\alpha_{r,\alpha }+\beta_{r,\alpha }}{A_{r,\alpha }}<1 \end{array}$$ (27)

*Proof*. (i) Implies that *α*, *β*, *A*, *x*_−_1 and $x_{0}\in R_{F}^{+}$. Then ∃ +ve number which belong to *R* i.e $M_{\alpha }^{\prime },N_{\alpha }^{\prime },M_{\beta }^{\prime },N_{\beta }^{\prime },M_{A}^{\prime },N_{A}^{\prime },M_{-}^{\prime }1,N_{-}^{\prime }1,M_{0}^{\prime },N_{0}$ such that:

$$\begin{array}{c} \left[\alpha_{l,\alpha },\alpha_{r,\alpha }\right]\subseteq \left[M_{\alpha }^{\prime },N_{\alpha }^{\prime }\right],\left[\beta_{l,\alpha },\beta_{r,\alpha }\right]\subseteq \left[M_{\beta }^{\prime },N_{\beta }^{\prime }\right],\left[A_{l,\alpha },A_{r,\alpha }\right]\subseteq \left[M_{A}^{\prime },N_{A}^{\prime }\right], \end{array}$$

$$\begin{array}{c} \left[L_{-}^{\prime }1,\alpha ,R_{-}^{\prime }1,\alpha \right]\subseteq \left[M_{-}^{\prime }1,N_{-}^{\prime }1\right],\left[L_{0}^{\prime },\alpha ,R_{0}^{\prime },\alpha \right]\subseteq \left[M_{0}^{\prime },N_{0}^{\prime }\right] \end{array}$$ (28)

Let the +ve results of 1 is *x*_*n*_. Now using 25, 28 and lemma 3.2, We get

$$\begin{array}{c} L_{n,\alpha }^{\prime }\geq \frac{\alpha_{l,\alpha }+\beta_{l,\alpha }log\frac{\alpha_{r,\alpha }+\beta_{r,\alpha }}{A_{r,\alpha }}}{A_{l,\alpha }+\frac{\alpha_{l,\alpha }+\beta_{l,\alpha }}{A_{l,\alpha }}}\geq \frac{M_{\alpha }^{\prime }+N_{\beta }^{\prime }log\frac{N_{\alpha }^{\prime }+N_{\beta }^{\prime }}{M_{A}^{\prime }}}{N_{A}^{\prime }+\frac{N_{\alpha }^{\prime }+N_{\beta }^{\prime }}{M_{A}^{\prime }}}=M^{\prime } \end{array}$$

$$\begin{array}{c} R_{n,\alpha }\leq \frac{\alpha_{r,\alpha }+\beta_{r,\alpha }}{A_{r,\alpha }}\leq \frac{N_{\alpha }^{\prime }+N_{\beta }^{\prime }}{M_{A}^{\prime }}=N^{\prime }. \end{array}$$ (29)

Which shows that $\left[L_{n.\alpha }^{\prime },R_{n,\alpha }^{\prime }\right]\subseteq \left[M^{\prime },N^{\prime }\right]$ is bounded and persistence.

Now for (ii) suppose the following systems

$$\begin{array}{c} L_{\alpha }^{\prime }=\frac{\alpha_{l,\alpha }+\beta_{l,\alpha }logR_{\alpha }^{\prime }}{A_{l,\alpha }+L_{\alpha }^{\prime }},R_{\alpha }^{\prime }=\frac{\alpha_{r,\alpha }+\beta_{r,\alpha }logL_{\alpha }^{\prime }}{A_{r,\alpha }+R_{\alpha }^{\prime }},\;0<\alpha \leq \;1. \end{array}$$ (30)

By preceding this

$$\begin{array}{c} \frac{\alpha_{l,\alpha }+\beta_{l,\alpha }log\frac{\alpha_{r,\alpha }+\beta_{r,\alpha }}{A_{r,\alpha }}}{A_{l,\alpha }+\frac{\alpha_{l,\alpha }+\beta_{l,\alpha }}{A_{l,\alpha }}}\leq L_{\alpha }\leq \frac{\alpha_{l,\alpha }+\beta_{l,\alpha }}{A_{l,\alpha }}.\frac{\alpha_{r,\alpha }+\beta_{r,\alpha }log\frac{\alpha_{l,\alpha }+\beta_{l,\alpha }}{A_{l,\alpha }}}{A_{l,\alpha }+\frac{\alpha_{r,\alpha }+\beta_{r,\alpha }}{A_{r,\alpha }}}\leq R_{\alpha }\leq \frac{\alpha_{r,\alpha }+\beta_{r,\alpha }}{A_{r,\alpha }} \end{array}$$ (31)

Let *x*_*n*_ be the fuzzy solution of 1 and [*x*_*n*_]_*α*_ = [*L*_*n*,*α*_, *R*_*n*,*α*_], Now from 25 we have

$$\begin{array}{c} L_{n+1,\alpha }^{\prime }=\frac{\alpha_{l,\alpha }+\beta_{l,\alpha }logR_{n}^{\prime },\alpha }{A_{l,\alpha }+L_{n-1,\alpha }^{\prime }},R_{n+1,\alpha }^{\prime }=\frac{\alpha_{r,\alpha }+\beta_{r,\alpha }logL_{n}^{\prime },\alpha }{A_{r,\alpha }+R_{n-1,\alpha }^{\prime }},\alpha \in \left(0,1\right]. \end{array}$$ (32)

By using 26, (27) and lemmas 3.2 and 3.3, It was shown that (32) has a unique +ve EP $\left[L_{\alpha }^{\prime },R_{\alpha }^{\prime }\right]$, when *α* ∈ (0, 1).

$$\begin{array}{c} \underset{n\to \infty }{\text{lim}}L_{n.\alpha }^{\prime }=L_{\alpha }^{\prime }\;and\;\underset{n\to \infty }{\text{lim}}R_{n.\alpha }^{\prime }=R_{\alpha }^{\prime } \end{array}$$ (33)

Now from 29 and 31 we have 0 < *α*_1_ < *α*_2_ < 1, Then

$$\begin{array}{c} 0<L_{\alpha_{1}}\leq L_{\alpha_{2}}^{\prime }\leq R_{\alpha_{2}}^{\prime }\leq R_{\alpha_{1}}^{\prime }<1 \end{array}$$ (34)

Since [*α*_*l*,*α*_, *α*_*r*,*α*_, *β*_*l*,*α*_, *β*_*r*,*α*_, *A*_*l*,*α*_, *A*_*r*,*α*_] be the left continuous, then from 30, we conclude that *L*_*α*_, *R*_*α*_ are also continuous from left side.

Now by using 31, we conclude that

$$\begin{array}{c} c=\frac{M_{\alpha }^{\prime }+M_{\beta }^{\prime }log\frac{N_{\alpha }^{\prime }+N_{\beta }^{\prime }}{N_{A}^{\prime }}}{N_{A}^{\prime }+\frac{N_{\alpha }^{\prime }+N_{\beta }^{\prime }}{M_{A}^{\prime }}}\leq \frac{\alpha_{l,\alpha }+\beta_{l,\alpha }log\frac{\alpha_{r,\alpha }+\beta_{r,\alpha }}{A_{r,\alpha }}}{A_{l,\alpha }+\frac{\alpha_{l,\alpha }+\beta_{l,\alpha }}{A_{l,\alpha }}}\leq L_{\alpha }\leq R_{\alpha }^{\prime }\leq \frac{N_{\alpha }^{\prime }+N_{\beta }^{\prime }}{M_{A}^{\prime }}=d \end{array}$$ (35)

Now from 35 if [*L*_*α*_, *R*_*α*_] ⊂ [*c*, *d*], and ∪_*α*∈(0,1]_[*L*_*α*_, *R*_*α*_] ⊂ [*c*, *d*], its clearly obtain the given results

$$\begin{array}{c} \cup_{\alpha \in (0,1]}\left[L_{\alpha }^{\prime },R_{\alpha }^{\prime }\right]\;is,compect,then\;\cup_{\alpha \in (0,1]}\left[L_{\alpha }^{\prime },R_{\alpha }^{\prime }\right]\subset \left(0,\infty \right). \end{array}$$ (36)

From definition 2.2, (30), 34 and 36 it is clear that there exist *x* ∈ *R*_*F*_, which satisfying

$$\begin{array}{c} x=\frac{\alpha +\beta logx}{A+x},{\left[x\right]}_{\alpha }=\left[L_{\alpha }^{\prime },R_{\alpha }^{\prime }\right],\alpha \in \left(0,1\right] \end{array}$$ (37)

If the Eq 1 has another +ve EP $\bar{x}$, then we have $\left[{\bar{L^{\prime }}}_{\alpha },{\bar{R^{\prime }}}_{\alpha }\right]:\left(0,1\right)\to \left(0,\infty \right)$ which satisfying:

$$\begin{array}{c} \bar{x}=\frac{\alpha +\beta log\bar{x}}{A+x},\;also\;{\left[\bar{x}\right]}_{\alpha }=\left[{\bar{L^{\prime }}}_{\alpha },{\bar{R^{\prime }}}_{\alpha }\right],\;\forall \;0<\alpha \leq 1 \end{array}$$

Where

$$\begin{array}{c} {\bar{L^{\prime }}}_{\alpha }=\frac{\alpha_{l,\alpha }+\beta_{l,\alpha }log{\bar{R^{\prime }}}_{\alpha }}{A_{l,\alpha }+{\bar{L^{\prime }}}_{\alpha }},{\bar{R^{\prime }}}_{\alpha }=\frac{\alpha_{r,\alpha }+\beta_{r,\alpha }log{\bar{L^{\prime }}}_{\alpha }}{A_{r,\alpha }+{\bar{R^{\prime }}}_{\alpha }},\;0<\alpha \leq 1. \end{array}$$

So $\bar{L^{\prime }}={Ł}_{\alpha }^{\prime }$ and $\bar{R^{\prime }}=R_{\alpha }^{\prime },and\;0<\alpha \leq 1$. We see that $\left[\bar{x}=x\right]$, which shows *x* has a unique +ve EP of Eq 1.

Now from (33), we deduct

$$\begin{array}{c} \underset{n\to \infty }{\text{lim}}D\left(x_{n},x\right)=\underset{n\to \infty }{\text{lim}}sup_{\alpha \in (0,1]}\{max|L_{n}^{\prime },\alpha -L_{\alpha }^{\prime }\left|,\right|R_{n}^{\prime },\alpha -R_{\alpha }^{\prime }|\}=0. \end{array}$$

It follows that all positive solutions *x*_*n*_ of 1 converge to a single +ve EP *x* as *n* → ∞.

Now if case (ii) is hold, i.e

$$\begin{array}{c} \frac{\alpha_{r,\alpha }+\beta_{r,\alpha }logL_{n}^{\prime },\alpha }{\alpha_{l,\alpha }+\beta_{l,\alpha }logR_{n}^{\prime },\alpha }\leq \frac{A_{r,\alpha }+R_{n-1,\alpha }^{\prime }}{A_{l,\alpha }+L_{n-1,\alpha }^{\prime }}\;for\;n\geq 0\;\alpha \in \left(0,1\right]\;forall\;n\in W,\alpha \in \left(0,1\right]. \end{array}$$

we have

$$\begin{array}{c} R_{n+1,\alpha }^{\prime }=\frac{\alpha_{l,\alpha }+\beta_{l,\alpha }logR_{n}^{\prime },\alpha }{A_{l,\alpha }+L_{n-1,\alpha }^{\prime }},L_{n+1,\alpha }^{\prime }=\frac{\alpha_{r,\alpha }+\beta_{r,\alpha }logL_{n}^{\prime },\alpha }{A_{r,\alpha }+R_{n-1,\alpha }^{\prime }},\alpha \in \left(0,1\right]. \end{array}$$ (38)

**Lemma 3.4**. *Let us Consider the system of DE*

$$\begin{array}{c} y_{n+1}=\frac{\alpha_{2}+\beta_{2}logy_{n}}{a_{2}+z_{n-1}},\;and\;z_{n+1}=\frac{\alpha_{1}+\beta_{1}logz_{n}}{a_{1}+y_{n-1}},n\in W. \end{array}$$ (39)

∀*α*_*i*_, *β*_*i*_, *a*_*i*_ ∈ (0, +∞) *where i* ∈ [1, 2] *the initial-values y*_−1_, *z*_−1_, *y*_0_, *z*_0_ ∈ *R*^+^. *Then the given two conditions hold*:

*(i) The results of* 39 *is persistence and bounded*.

*(ii) The systems* 39 *have unique +ve EP*.

$$\begin{array}{c} \left(\bar{y},\bar{z}\right)\in \left[P_{1}=\frac{\alpha_{2}+\beta_{2}log\frac{\alpha_{2}+\beta_{2}}{a_{2}}}{a_{2}+\frac{\alpha_{1}+\beta_{1}}{a_{1}}},Q_{1}=\frac{\alpha_{2}+\beta_{2}}{a_{2}}\right]\times \left[P_{2}=\frac{\alpha_{1}+\beta_{1}log\frac{\alpha_{1}+\beta_{1}}{a_{1}}}{a_{1}+\frac{\alpha_{2}+\beta_{2}}{a_{2}}},Q_{2}=\frac{\alpha_{1}+\beta_{1}}{a_{1}}\right] \end{array}$$

*if*

$$\begin{array}{c} \left(\left(Q_{2}+1\right)\beta_{1}logP_{2}+\alpha_{1}\right)\left(\left(\Gamma -1\right)\beta_{2}log\Gamma +\alpha_{2}\right)<P_{2}^{2}\Gamma^{2} \end{array}$$ (40)

*and*

$$\begin{array}{c} \left(\left(Q_{1}+1\right)\beta_{2}logP_{1}+\alpha_{2}\right)\left(\left(\gamma -1\right)\beta_{1}log\gamma +\alpha_{1}\right)<P_{1}^{2}\gamma^{2} \end{array}$$ (41)

*Where*

$$\begin{array}{c} \Gamma =-\left(\frac{\alpha_{1}+\beta_{1}logQ_{2}}{Q_{2}}-a_{1}\right),\gamma =-\left(\frac{\alpha_{2}+\beta_{2}logQ_{1}}{Q_{1}}-a_{2}\right) \end{array}$$

*Proof*. Now condition (i) is clear from (39) i.e:

$$\begin{array}{c} y_{n}\leq \frac{\alpha_{2}+\beta_{2}}{a_{2}}=Q_{1},z_{n}\leq \frac{\alpha_{1}+\beta_{1}}{a_{1}}=Q_{2} \end{array}$$ (42)

By 39 and 42 we have

$$\begin{array}{c} y_{n}\geq \frac{\alpha_{2}+\beta_{2}log\frac{\alpha_{2}+\beta_{2}}{a_{2}}}{a_{2}+\frac{\alpha_{1}+\beta_{1}}{a_{1}}}=P_{1},z_{n}\geq \frac{\alpha_{1}+\beta_{1}log\frac{\alpha_{1}+\beta_{1}}{a_{1}}}{a_{1}+\frac{\alpha_{2}+\beta_{2}}{a_{2}}}=P_{2} \end{array}$$ (43)

Now from Eqs 42 and 43 we observe that the Equation (3.37) is bounded and persistence i.e

$$\begin{array}{c} P_{1}\leq y_{n}\leq Q_{1},\;and\;P_{2}\leq z_{n}\leq Q_{2},n\geq 1. \end{array}$$

(ii) implies that we consider

$$\begin{array}{c} y=\frac{\alpha_{2}+\beta_{2}logy}{a_{2}+z},z=\frac{\alpha_{1}+\beta_{1}logz}{a_{1}+y} \end{array}$$ (44)

Eq 44 can also be written as

$$\begin{array}{c} z=\frac{\alpha_{2}+\beta_{2}logy-a_{2}y}{y},\;y=\frac{\alpha_{1}+\beta_{1}logz-a_{1}z}{z} \end{array}$$ (45)

Now from 45 we obtain

$$\begin{array}{c} F\left(z\right)=\frac{\alpha_{2}+\beta_{2}logg(z)}{g(z)}-a_{2}-z, \end{array}$$ (46)

Where

$$\begin{array}{c} y=g\left(z\right)=\frac{\alpha_{1}+\beta_{1}logz-a_{1}z}{z},z\in \left[P_{2}=\frac{\alpha_{1}+\beta_{1}log\frac{\alpha_{1}+\beta_{1}}{a_{1}}}{a_{1}+\frac{\alpha_{2}+\beta_{2}}{a_{2}}},Q_{2}=\frac{\alpha_{1}+\beta_{1}}{a_{1}}\right], \end{array}$$ (47)

we have

$$\begin{array}{c} F\left(z\right):\left[P_{2}=\frac{\alpha_{1}+\beta_{1}log\frac{\alpha_{1}+\beta_{1}}{a_{1}}}{a_{1}+\frac{\alpha_{2}+\beta_{2}}{a_{2}}},Q_{2}=\frac{\alpha_{1}+\beta_{1}}{a_{1}}\right]\to \left[P_{2}=\frac{\alpha_{1}+\beta_{1}log\frac{\alpha_{1}+\beta_{1}}{a_{1}}}{a_{1}+\frac{\alpha_{2}+\beta_{2}}{a_{2}}},Q_{2}=\frac{\alpha_{1}+\beta_{1}}{a_{1}}\right] \end{array}$$

By following 46, 47 we get

$$\begin{array}{c} F^{\prime }\left(z\right)=-g^{\prime }\left(z\right)\frac{\beta_{2}g\left(z\right)logg(z)+\alpha_{2}+\beta_{2}logg(z)}{g^{2}\left(z\right)}-1, \end{array}$$ (48)

$$g^{\prime }\left(z\right)=-\frac{\beta_{1}zlogz+\alpha_{1}+\beta_{1}logz}{z^{2}}$$ (49)

Now let

$$\begin{array}{c} \bar{z}\in \left[P_{2}=\frac{\alpha_{1}+\beta_{1}log\frac{\alpha_{1}+\beta_{1}}{a_{1}}}{a_{1}+\frac{\alpha_{2}+\beta_{2}}{a_{2}}},Q_{2}=\frac{\alpha_{1}+\beta_{1}}{a_{1}}\right] \end{array}$$

be the results of F(z)=0 and then by following 45 and 46

$$\begin{array}{c} \alpha_{2}+\beta_{2}logg(\bar{z})=g\left(\bar{z}\right)\left(a_{2}+\bar{z}\right) \end{array}$$ (50)

Where

$$\begin{array}{c} g\left(\bar{z}\right)=\frac{\alpha_{1}+\beta_{1}log\bar{z}}{\bar{z}}-a_{1}. \end{array}$$ (51)

By putting 48, 50, 51, in Eq 48 we have

$$\begin{array}{c} F^{\prime }\left(z\right)=-\frac{\beta_{1}\bar{z}log\bar{z}+\alpha_{1}+\beta_{1}log\bar{z}}{{\bar{z}}^{2}}\times \frac{\beta_{1}(\left(\frac{\alpha_{1}+\beta_{1}log\bar{z}}{\bar{z}}-a_{1}\right)log\left(\frac{\alpha_{1}+\beta_{1}log\bar{z}}{\bar{z}}-a_{1}\right)+\alpha_{2}+\beta_{2}log\left(\frac{\alpha_{1}+\beta_{1}log\bar{z}}{\bar{z}}-a_{1}\right)}{{\left(\frac{\alpha_{1}+\beta_{1}log\bar{z}}{\bar{z}}-a_{1}\right)}^{2}}-1 \end{array}$$ (52)

Now proceeding 41 by putting the value of Γ, we have

$$\begin{array}{c} F^{\prime }\left(z\right)\leq \frac{\left(\left(Q_{2}+1\right)\beta_{1}log\;P_{2}+\alpha_{1}\right)\left(\left(-\left(\frac{\alpha_{1}+\beta_{1}logQ_{2}}{Q_{2}}-a_{1}\right)-1\right)\beta_{2}e^{-(\frac{\alpha_{1}+\beta_{1}logQ_{2}}{Q_{2}}-a_{1})}+\alpha_{2}\right)}{P_{2}^{2}{\left(\frac{\alpha_{1}+\beta_{1}logQ_{2}}{Q_{2}}-a_{1}\right)}^{2}}-1 \end{array}$$

$$\begin{array}{c} =\frac{\left(\left(Q_{2}+1\right)\beta_{1}log\;P_{2}+\alpha_{1}\right)\left(\left(\Gamma -1\right)\beta_{2}log\Gamma +\alpha_{2}\right)}{P_{2}^{2}\Gamma^{2}}-1<0 \end{array}$$ (53)

Therefore F(z)=0 get unique EP i.e

$$\begin{array}{c} \bar{z}\in \left[P_{2}=\frac{\alpha_{1}+\beta_{1}log\frac{\alpha_{1}+\beta_{1}}{a_{1}}}{a_{1}+\frac{\alpha_{2}+\beta_{2}}{a_{2}}},Q_{2}=\frac{\alpha_{1}+\beta_{1}}{a_{1}}\right]. \end{array}$$

The component $\bar{y}\in \left[P_{1}=\frac{\alpha_{2}+\beta_{2}log\frac{\alpha_{2}+\beta_{2}}{a_{2}}}{a_{2}+\frac{\alpha_{1}+\beta_{1}}{a_{1}}},Q_{1}=\frac{\alpha_{2}+\beta_{2}}{a_{2}}\right]$ can also be obtain by similar arguments. This is the required proof of (ii).

**Lemma 3.5**. *Now for +ve EP* $\left(\bar{y},\bar{z}\right)$ *of* 39 *can be considered stable locally asymptotically if*:

$$\begin{array}{c} =\frac{\beta_{2}}{\left(P_{1}\left(a_{2}+P_{2}\right)\right)}+\frac{\beta_{1}}{\left(P_{2}\left(a_{1}+P_{1}\right)\right)}+\frac{\beta_{2}}{\left(P_{1}\left(a_{2}+P_{2}\right)\right)}\frac{\beta_{1}}{\left(P_{2}\left(a_{1}+P_{1}\right)\right)}+\frac{\alpha_{2}+\beta_{2}logP_{1}}{{\left(a_{2}+P_{2}\right)}^{2}}\frac{\alpha_{1}+\beta_{1}logP_{2}}{{\left(a_{1}+P_{1}\right)}^{2}}<1 \end{array}$$ (54)

*Proof*. The Jacobian matrix $J(\bar{y},\bar{z})$ of 39 has the value of $\left(\bar{y},\bar{z}\right)$ being the unique equilibrium of 39:

$$\begin{array}{c} J_{\left(\bar{y},\bar{z}\right)}=[\begin{array}{cccc} D_{1} & 0 & 0 & D_{2}\\ 1 & 0 & 0 & 0\\ 0 & E_{1} & E_{2} & 0\\ 0 & 0 & 1 & 0 \end{array}] \end{array}$$

Where

$D_{1}=\frac{\beta_{2}}{\left(\bar{y}\left(a_{2}+\bar{z}\right)\right)}$ , $D_{2}=-\frac{\alpha_{2}+\beta_{2}log\bar{y}}{{\left(a_{2}+\bar{z}\right)}^{2}}$,$E_{1}=-\frac{\alpha_{1}+\beta_{1}log\bar{z}}{{\left(a_{1}+\bar{y}\right)}^{2}}$, $E_{2}=\frac{\beta_{1}}{\left(\bar{z}\left(a_{1}+\bar{y}\right)\right)}$

And the characteristics equations of $J(\bar{y},\bar{z})$, at $\left(\bar{y},\bar{z}\right)$ is given by:

$$\begin{array}{c} \lambda^{4}+\mu_{1}\lambda^{3}+\mu_{2}\lambda^{2}+\mu_{3}=0. \end{array}$$ (55)

where *μ*_1_ = −(*D*_1_ + *E*_2_), *μ*_2_ = *D*_1_*E*_2_, *μ*_3_ = −*D*_2_*E*_1_

Since condition 54 hold i.e

$$\begin{array}{c} \sum_{n=1}^{3}|\mu_{i}\left|=\right|\mu_{1}\left|+\right|\mu_{2}\left|+\right|\mu_{3}| \end{array}$$

By putting the values of *μ*_1_, *μ*_2_, *and μ*_3_ we have,

$$\begin{array}{c} \sum_{n=1}^{3}|\mu_{i}\left|=\right|\frac{\beta_{2}}{\left(\bar{y}\left(a_{2}+\bar{z}\right)\right)}+\frac{\beta_{1}}{\left(\bar{z}\left(a_{1}+\bar{y}\right)\right)}\left|+\right|\frac{\beta_{2}}{\left(\bar{y}\left(a_{2}+\bar{z}\right)\right)}\times \frac{\beta_{1}}{\left(\bar{z}\left(a_{1}+\bar{y}\right)\right)}\left|+\right|\frac{\alpha_{2}+\beta_{2}log\bar{y}}{{\left(a_{2}+\bar{z}\right)}^{2}}\times \frac{\alpha_{1}+\beta_{1}log\bar{z}}{{\left(a_{1}+\bar{y}\right)}^{2}}|\\ \leq |\frac{\beta_{2}}{\left(P_{1}\left(a_{2}+P_{2}\right)\right)}+\frac{\beta_{1}}{\left(P_{2}\left(a_{1}+P_{1}\right)\right)}\left|+\right|\frac{\beta_{2}}{\left(P_{1}\left(a_{2}+P_{2}\right)\right)}\times \frac{\beta_{1}}{\left(P_{2}\left(a_{1}+P_{1}\right)\right)}\left|+\right|\frac{\alpha_{2}+\beta_{2}logP_{1}}{{\left(a_{2}+P_{2}\right)}^{2}}\times \frac{\alpha_{1}+\beta_{1}logP_{2}}{{\left(a_{1}+P_{1}\right)}^{2}}| \end{array}$$

$$\begin{array}{c} =\frac{\beta_{2}}{\left(P_{1}\left(a_{2}+P_{2}\right)\right)}+\frac{\beta_{1}}{\left(P_{2}\left(a_{1}+P_{1}\right)\right)}+\frac{\beta_{2}}{\left(P_{1}\left(a_{2}+P_{2}\right)\right)}\frac{\beta_{1}}{\left(P_{2}\left(a_{1}+P_{1}\right)\right)}+\frac{\alpha_{2}+\beta_{2}logP_{1}}{{\left(a_{2}+P_{2}\right)}^{2}}\frac{\alpha_{1}+\beta_{1}logP_{2}}{{\left(a_{1}+P_{1}\right)}^{2}}<1. \end{array}$$ (56)

56 implies that modules of 56 i.e |λ_*n*_| < 1. This means that $\left(\bar{y},\bar{z}\right)$ is locally asymptotically stable.

**Theorem 3.3**. *Consider a FDE* 1, *If*

$$\begin{array}{c} \frac{\alpha_{r,\alpha }+\beta_{r,\alpha }\times logL_{n,\alpha }}{\alpha_{l,\alpha }+\beta_{l,\alpha }\times logR_{n,\alpha }}\leq \frac{A_{r,\alpha }+R_{n-1,\alpha }}{A_{l,\alpha }+L_{n-1,\alpha }},\forall \;n\in Wand\;0<\alpha \leq 1 \end{array}$$ (57)

*Then the following results hold for two conditions i.e*

*(i) Every +ve results of* 1 *is exist and it is bounded*.

*(ii) The* Eq 1 *converges to a unique +ve EP x as n* → ∞ *and if* 0 < *α* ≤ 1, *then*

$$\begin{array}{c} (\left(Q_{1,\alpha }+1\right)\left(\beta_{r,\alpha }log\;P_{1,\alpha }+\alpha_{r,\alpha }\right)\left(\left(\gamma_{\alpha }-1\right)\beta_{l,\alpha }log\gamma_{\alpha }+\alpha_{l,\alpha }\right)<P_{1,\alpha }^{2}\left(\gamma_{\alpha }^{2}\right). \end{array}$$ (58)

*and*

$$\begin{array}{c} \left(Q_{2,\alpha }+1\right)\left(\beta_{l,\alpha }logP_{2,\alpha }+\alpha_{l\alpha }\right)\left(\left(\Gamma_{\alpha -1}\right)\beta_{r,\alpha }log\Gamma_{\alpha }+\alpha_{r,\alpha }\right)<P_{2,\alpha }^{2}\left(\Gamma_{\alpha }^{2}\right). \end{array}$$ (59)

*where*

$$\begin{array}{c} Q_{1,\alpha }=\frac{\alpha_{r,\alpha }+\beta_{r,\alpha }}{a_{r,\alpha }},\;Q_{2,\alpha }=\frac{\alpha_{l,\alpha }+\beta_{l,\alpha }}{a_{l,\alpha }},\;P_{1,\alpha }=\frac{\alpha_{r,\alpha }+\beta_{r,\alpha }logQ_{1,\alpha }}{a_{r,\alpha }+Q_{2,\alpha }},\;P_{2,\alpha }=\frac{\alpha_{l,\alpha }+\beta_{l,\alpha }logQ_{2,\alpha }}{a_{l,\alpha }+Q_{1,\alpha }} \end{array}$$

$$\begin{array}{c} \Gamma_{\alpha }=-\left(\frac{\alpha_{l,\alpha }+\beta_{l,\alpha }logQ_{2,\alpha }}{Q_{2,\alpha }}-a_{l,\alpha }\right),\gamma_{\alpha }=-\left(\frac{\alpha_{r,\alpha }+\beta_{r,\alpha }logQ_{1,\alpha }}{Q_{1,\alpha }}-a_{r,\alpha }\right). \end{array}$$

*Proof*. Condition (i) is similar to one of the previous assertion of the theorems (3.2) we suppose that *x*_*n*_ be the +ve results of (1.1), then from (3.36), (3.55) and lemma (3.4) then we have

$$\begin{array}{c} L_{n,\alpha }\geq \frac{\alpha_{l,\alpha }+\beta_{l,\alpha }log\frac{\alpha_{r,\alpha }+\beta_{r,\alpha }}{A_{r,\alpha }}}{A_{l,\alpha }+\frac{\alpha_{l,\alpha }+\beta_{l,\alpha }}{A_{l,\alpha }}}\geq \frac{M_{\alpha }+M_{\beta }log\frac{N_{\alpha }+N_{\beta }}{N_{A}}}{N_{A}+\frac{N_{\alpha }+N_{\beta }}{M_{A}}}=K,\;R_{n,\alpha }\leq \frac{\alpha_{l,\alpha }+\beta_{l,\alpha }}{A_{l,\alpha }}\leq \frac{N_{\alpha }+N\beta }{M_{A}}=L \end{array}$$ (60)

Thus, [*L*_*n*,*α*_, *R*_*n*,*α*_] ⊆ [*K*, *L*], which shows that it is bounded.

(ii) The proof is same as we have done Theorem 25.

## 4 Numerical example

Now we discuss some examples to verifying our results. Consider the third order exponential type FDE.

**Example 4.1**.

$$\begin{array}{c} x_{n+1}=\frac{\alpha +\beta logx_{n}}{A+x_{n-1}},\;n\in W \end{array}$$ (61)

*Where α*, *β and A be the triangular fuzzy number and x*_−1_, *x*_0_ *be the initial condition i.e*

$$\begin{array}{c} \alpha \left(x\right)=\{\begin{array}{cc} x-1 & if\;x\in [1,2]\\ -x+3 & if\;x\in [2,3] \end{array},\beta \left(x\right)=\{\begin{array}{cc} 2x-1 & if\;x\in [0.5,1]\\ -2x+5 & if\;x\in [1,2] \end{array} \end{array}$$ (62)

$$\begin{array}{c} A\left(x\right)=\{\begin{array}{cc} 4x-6 & if\;1.5\leq x\leq 1.75\\ -4x+8 & if\;1.75\leq x\leq 2 \end{array},x_{0}\left(x\right)=\{\begin{array}{cc} x-7 & if\;7\leq x\leq 8\\ -x+9 & if\;8\leq x\leq 9 \end{array} \end{array}$$ (63)

$$\begin{array}{c} x_{-1}\left(x\right)=\{\begin{array}{cc} x-10 & if\;x\in [10,11]\\ -x+12 & if\;x\in [11,12] \end{array} \end{array}$$ (64)

*Now from* (4.2), *we get*

$$\begin{array}{c} {\left[\alpha \right]}_{\alpha }=\left[1+\alpha ,3-\alpha \right],\;{\left[\beta \right]}_{\alpha }=\left[0.5+0.5\alpha ,2-\alpha \right] \end{array}$$ (65)

*Also from* (4.3), *we have*

$$\begin{array}{c} {\left[A\left(x\right)\right]}_{\alpha }=\left[1.5+0.25\alpha ,2-0.25\alpha \right],\;{\left[x_{0}\right]}_{\alpha }=\left[7+\alpha ,8-\alpha \right] \end{array}$$ (66)

*And last we take* (4.4), *and get*

$$\begin{array}{c} {\left[x_{-1}\right]}_{\alpha }=\left[10+\alpha ,12-\alpha \right] \end{array}$$ (67)

*Therefore, by following the above example and have*

$$\begin{array}{c} \bar{\cup_{\alpha \in (0,1]}{\left[\beta \right]}_{\alpha }}=\left[0.5,2\right],\;\bar{\cup_{\alpha \in (0,1]}{\left[\alpha \right]}_{\alpha }}=\left[1,3\right] \end{array}$$

$$\begin{array}{c} \bar{\cup_{\alpha \in (0,1]}{\left[A\left(x\right)\right]}_{\alpha }}=\left[0.5,2\right],\;\bar{\cup_{\alpha \in (0,1]}{\left[x_{0}\right]}_{\alpha }}=\left[7,9\right] \end{array}$$

$$\begin{array}{c} \bar{\cup_{\alpha \in (0,1]}{\left[x_{-1}\right]}_{\alpha }}=\left[10,12\right] \end{array}$$

*Now by consider the DE from* 81 *when α cut is apply then we consider a couple of DE with parameter α which are given by*:

$$\begin{array}{cc} L_{n+1,\alpha } & =\frac{\alpha_{l,\alpha }+\beta_{l,\alpha }\times logR_{n,\alpha }}{A_{l,\alpha }+L_{n-1,\alpha }},\;R_{n+1,\alpha }\frac{\alpha_{r,\alpha }+\beta_{r,\alpha }\times logL_{n,\alpha }}{A_{r,\alpha }+R_{n-1,\alpha }},\;0<\alpha \leq 1 \end{array}$$ (68)

**Fig 1:**
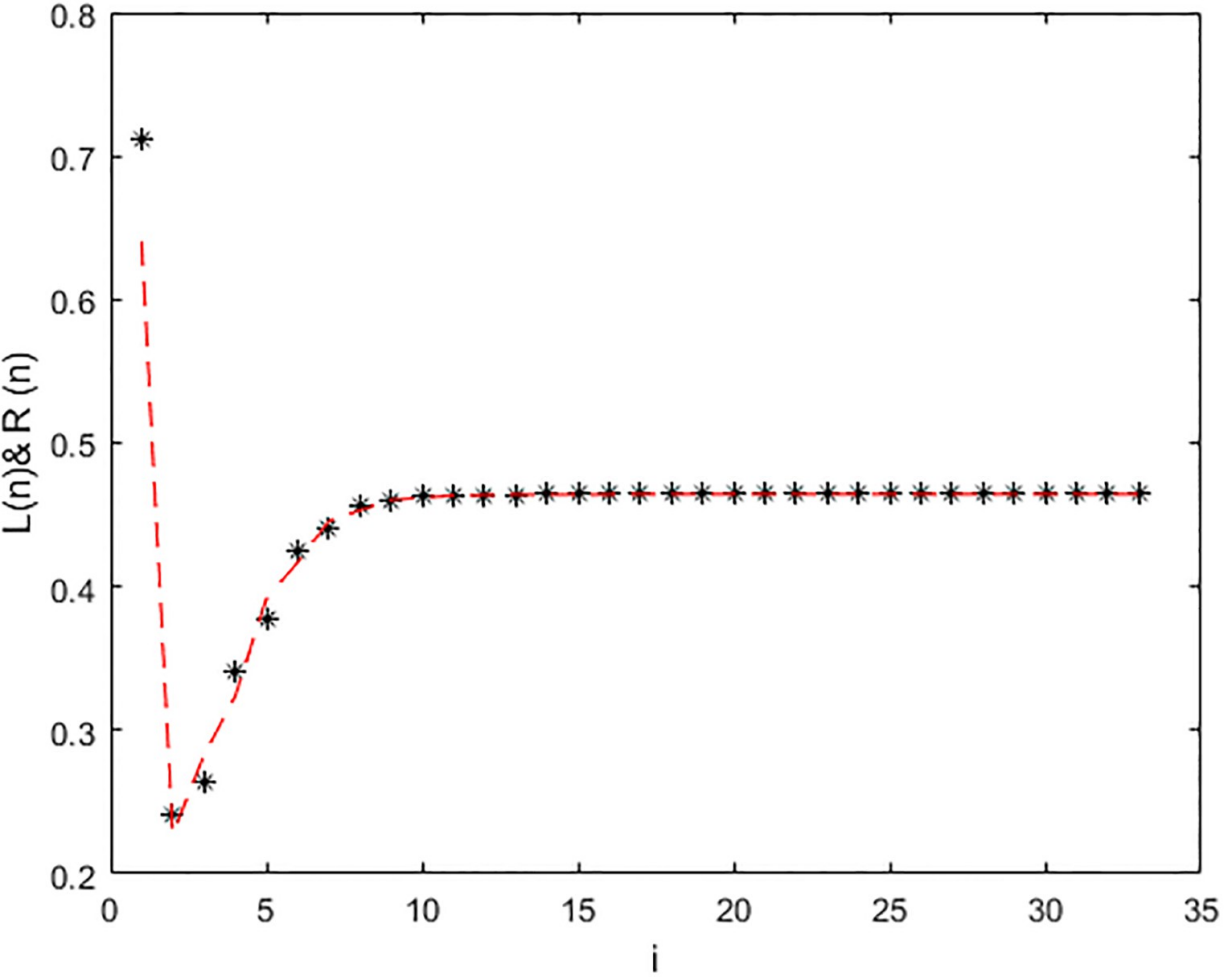
Solution of 81 at *α* = 0.

**Fig 2:**
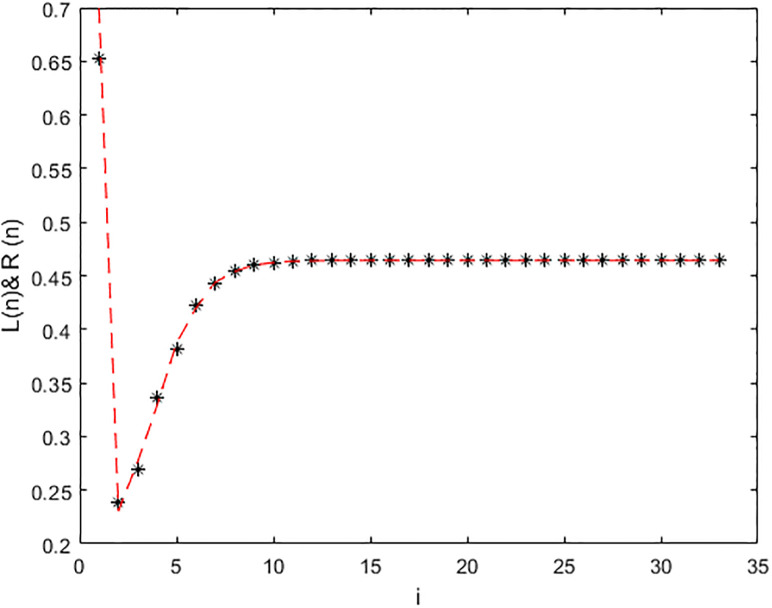
Solution of 81 at *α* = 1.

*In the* Figs 1, 2 and 5, *it shows that all required condition of theorems* 57 *holds, so every +ve solution x*_*n*_ *of* Eq 81 *is exists and bounded, also from theorems* 57, *and* Eq 81 *has a unique +ve (EP)* $\bar{x}=\left(0.442,0.4642,0.4642\right)$. *Moreover every +ve results x*_*n*_ *of* (4.1) *tends to* $\bar{x}$ *when i* → ∞ *see* Figs 1 and 2. *From* Fig 2 *we see that the +ve results x*_*n*_, [*x*]_*α*_ = [*L*_*n*,*α*_, *R*_*n*,*α*_], *of* Eq 81, *with initial-condition x*_−1_ = (10, 11, 12), *x*_0_ = (7, 8, 9) *which converge to a +ve (EP)* $\bar{x}=(\left(0.442,0.4642,0.4642\right)$ *as n* → ∞.

**Example 4.2**.

$$\begin{array}{c} x_{n+1}=\frac{\alpha +\beta logx_{n}}{A+x_{n-1}},\;n\in W \end{array}$$ (69)

*Where α*, *β and A be the triangular fuzzy number and x*_−1_, *x*_0_ *be the initial condition i.e*

$$\begin{array}{c} \alpha \left(x\right)=\{\begin{array}{cc} 2x-12 & if\;x\in [6,6.5]\\ -2x+14 & if\;x\in [6.5,7] \end{array},\beta \left(x\right)=\{\begin{array}{cc} 4x-12 & if\;x\in [3,3.25]\\ -4x+14 & if\;x\in [3.25,3.5] \end{array} \end{array}$$ (70)

$$\begin{array}{c} A\left(x\right)=\{\begin{array}{cc} 4x-15 & if\;\text{3}.75\leq x\leq 4\\ -4x+17 & if\;\text{4}\leq x\leq 4.25 \end{array},x_{0}\left(x\right)=\{\begin{array}{cc} 5x-18 & if\;\text{3}.6\leq x\leq 3.8\\ -5x+20 & if\;\text{3}.8\leq x\leq 4 \end{array} \end{array}$$ (71)

$$\begin{array}{c} x_{-1}\left(x\right)=\{\begin{array}{cc} 8x-18 & if\;x\in [2.25,2.375]\\ -8x+20 & if\;x\in [2.375,2.5] \end{array} \end{array}$$ (72)

*Now from* (4.10), *we get*

$$\begin{array}{c} {\left[\alpha \right]}_{\alpha }=\left[6+0.5\alpha ,7-0.5\alpha \right],\;{\left[\beta \right]}_{\alpha }=\left[3+0.25\alpha ,3.5-0.25\alpha \right] \end{array}$$ (73)

*Also from* (4.11), *we have*

$$\begin{array}{c} {\left[A\left(x\right)\right]}_{\alpha }=\left[3.75+0.25\alpha ,4.25-0.25\alpha \right],\;{\left[x_{0}\right]}_{\alpha }=\left[3.6+0.2\alpha ,4-0.2\alpha \right] \end{array}$$ (74)

*And last we take* (4.12), *and get*

$$\begin{array}{c} {\left[x_{-1}\right]}_{\alpha }=\left[2.25+0.125\alpha ,2.5-0.125\alpha \right] \end{array}$$ (75)

*Therefore, by following the above example and have*

$$\begin{array}{c} \bar{\cup_{\alpha \in (0,1]}{\left[\beta \right]}_{\alpha }}=\left[3.3,3.5\right],\;\bar{\cup_{\alpha \in (0,1]}{\left[\alpha \right]}_{\alpha }}=\left[6,7\right] \end{array}$$

$$\begin{array}{c} \bar{\cup_{\alpha \in (0,1]}{\left[A\left(x\right)\right]}_{\alpha }}=\left[3.75,4.25\right],\;\bar{\cup_{\alpha \in (0,1]}{\left[x_{0}\right]}_{\alpha }}=\left[3.6,4\right] \end{array}$$

$$\begin{array}{c} \bar{\cup_{\alpha \in (0,1]}{\left[x_{-1}\right]}_{\alpha }}=\left[2.25,2.5\right] \end{array}$$

*Now by consider the DE from* 69 *when α cut is apply then we consider a couple of DE with parameter α which are given by*:

$$\begin{array}{cc} L_{n+1,\alpha } & =\frac{\alpha_{l,\alpha }+\beta_{l,\alpha }\times logR_{n,\alpha }}{A_{l,\alpha }+L_{n-1,\alpha }},\;R_{n+1,\alpha }\frac{\alpha_{r,\alpha }+\beta_{r,\alpha }\times logL_{n,\alpha }}{A_{r,\alpha }+R_{n-1,\alpha }},\;0<\alpha \leq 1 \end{array}$$ (76)

**Fig 3:**
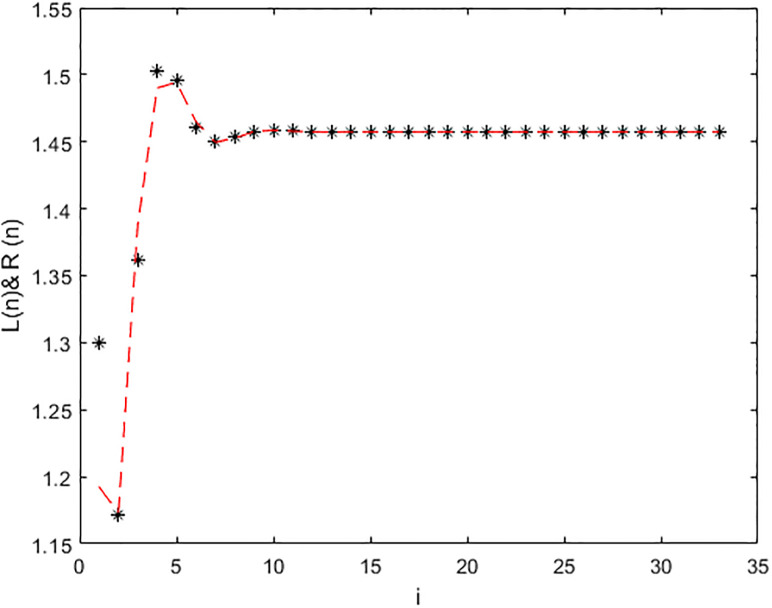
Solution of 69 at *α* = 0.

**Fig 4:**
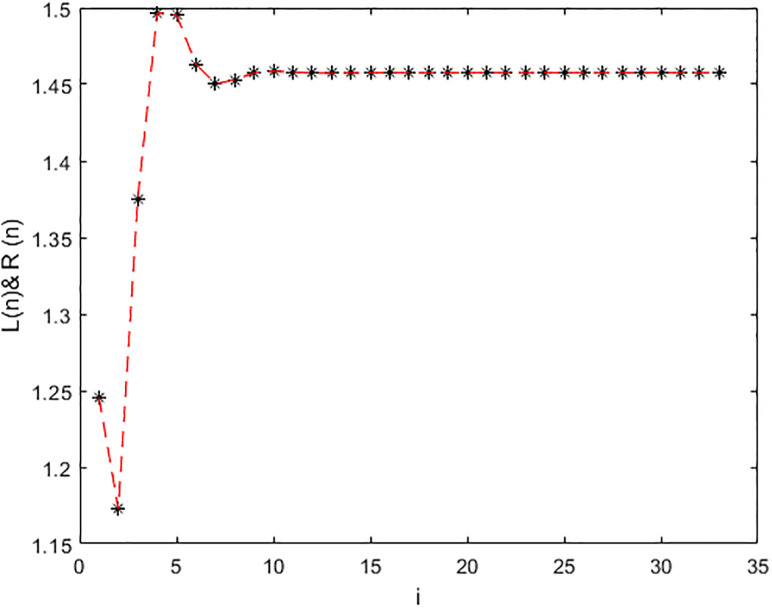
Solution of 69 at *α* = 1.

**Fig 5:**
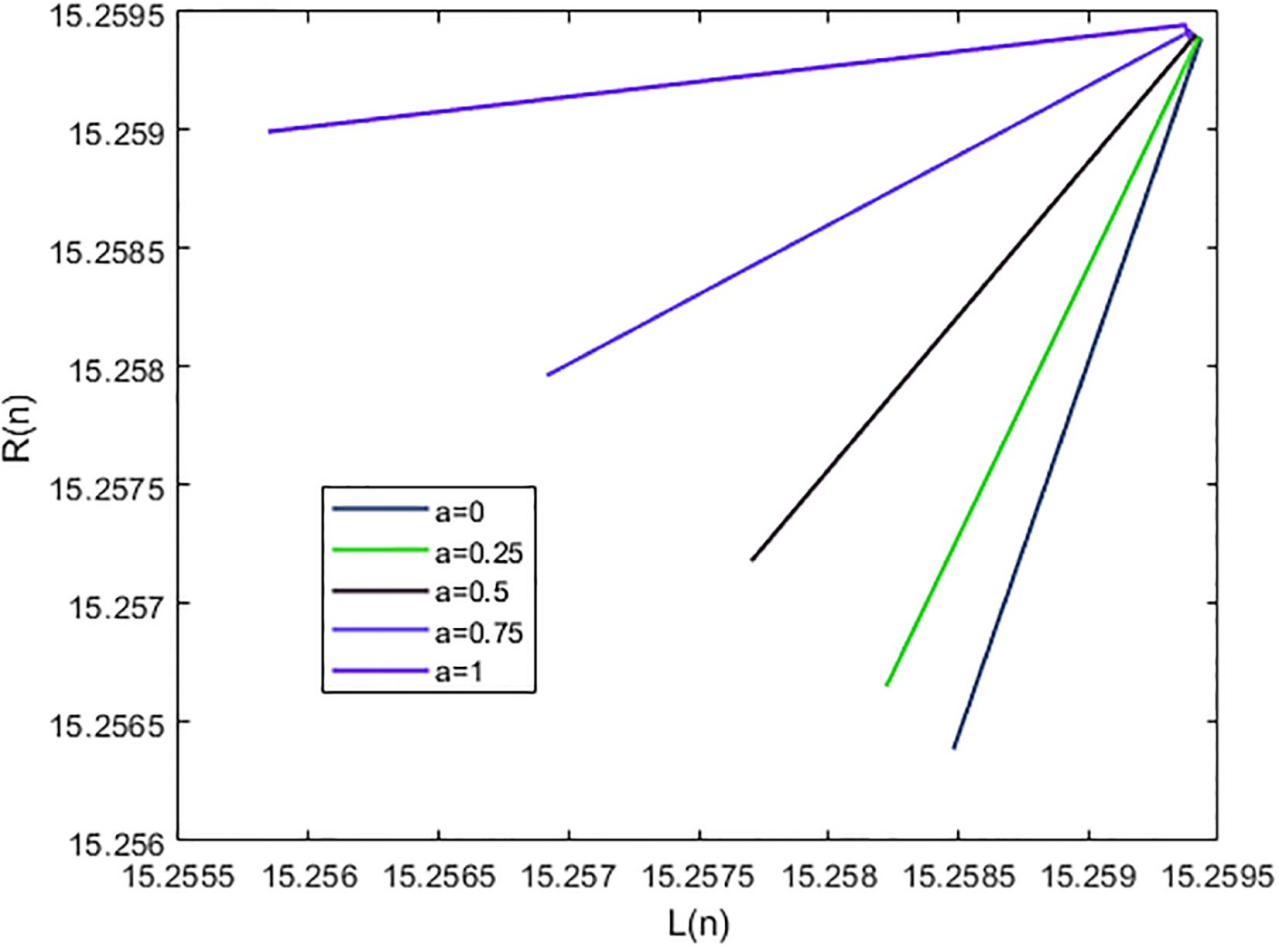
Solution of 69 at *α* = 0.

**Fig 6:**
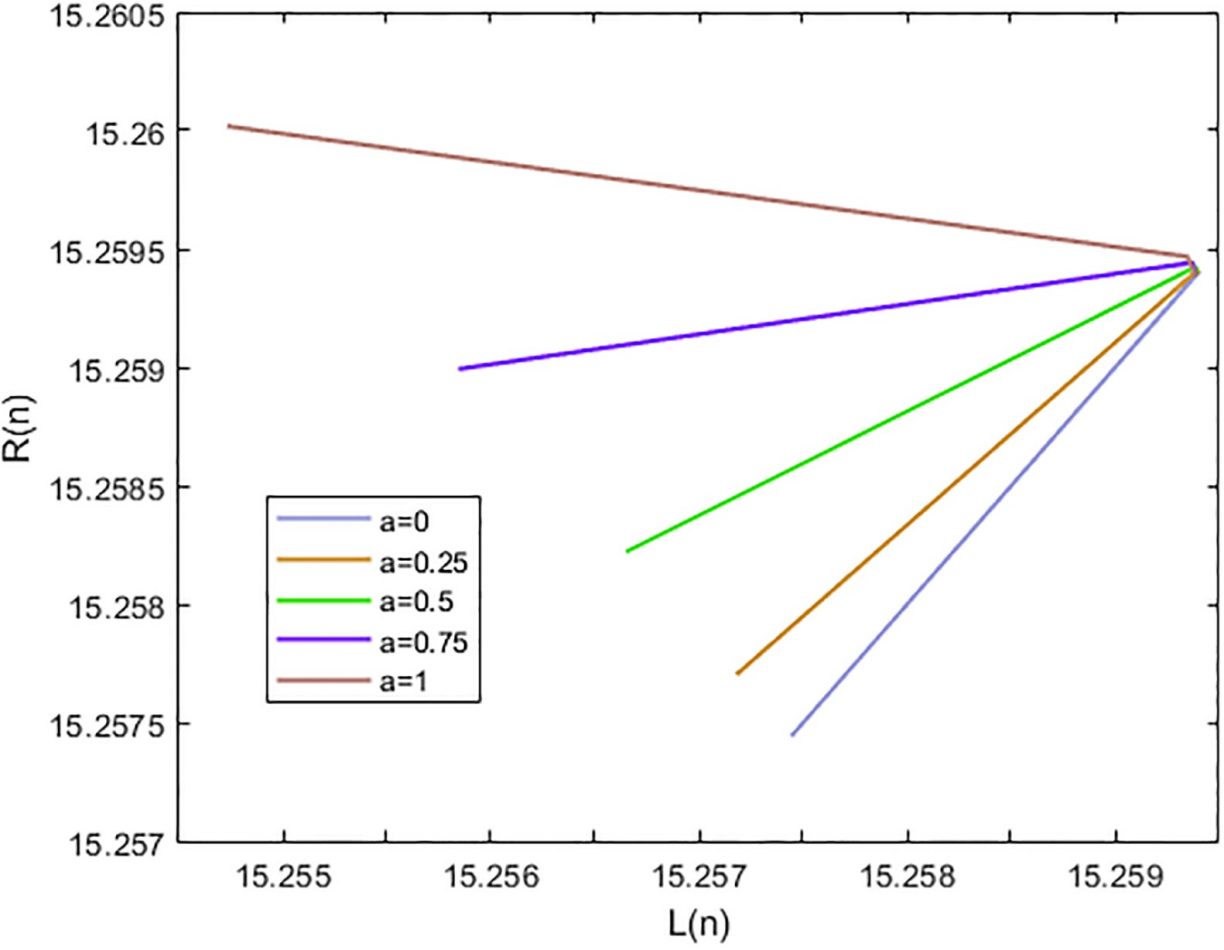
Solution of 69 at *α* = 1.

*In the* Figs 3–6, *its shows that all required condition of theorems* 57 *holds true, so every +ve solution x*_*n*_ *of* Eq 69 *is exists and bounded, also from theorems* 57, *and* Eq 69 *has a unique +ve (EP)* $\bar{x}=\left(1.4474,1.4574,1.4574\right)$. *Moreover every +ve results x*_*n*_ *of* 69 *tends to* $\bar{x}$ *when i* → ∞ *see* Figs 3–6. *From* Fig 3 *we see that the +ve results x*_*n*_, [*x*]_*α*_ = [*L*_*n*,*α*_, *R*_*n*,*α*_], *of* Eq 69, *with initial-condition x*_−1_ = (2.25, 2.375, 2.5), *x*_0_ = (3.6, 3.8, 4) *which converge to a +ve (EP)* $\bar{x}=\left(1.4474,1.4574,1.4574\right)$ *as n* → ∞.

**Example 4.3**.

$$\begin{array}{c} x_{n+1}=\frac{\alpha +\beta logx_{n}}{A+x_{n-1}},\;n\in W \end{array}$$ (77)

*Where α, β and A be the triangular fuzzy number and x*_−1_, *x*_0_ *be the initial condition that is if we take the crisps equations of this difference equation like as*;

$$\begin{array}{c} y_{n+1}=\frac{\alpha_{1}+\beta_{1}\times logz_{n}}{a_{1}+y_{n-1}},\;z_{n+1}=\frac{\alpha_{2}+\beta_{2}\times logy_{n}}{a_{1}+z_{n-1}},\;n\in W \end{array}$$ (78)

*then we consider the initial condition i.e x(1) = 2 y(1) = 5, x(2) = 7, y(2) = 1 and x(1) = 8 y(1) = 2, x(2) = 5, y(2) = 7 then the stability of the equations are shown below*:

**Example 4.4**.

$$\begin{array}{c} x_{n+1}=\frac{\alpha +\beta logx_{n}}{A+x_{n-1}},\;n\in W, \end{array}$$ (79)

*where α, β and A be the triangular fuzzy number and x*_−1_, *x*_0_ *be the initial condition that is if we take the crisps equations of this difference equation like as*;

$$\begin{array}{c} y_{n+1}=\frac{30+15\times logz_{n}}{15+y_{n-1}},\;z_{n+1}=\frac{25+15\times logy_{n}}{13+z_{n-1}},\;n\in W \end{array}$$ (80)

**Fig 7:**
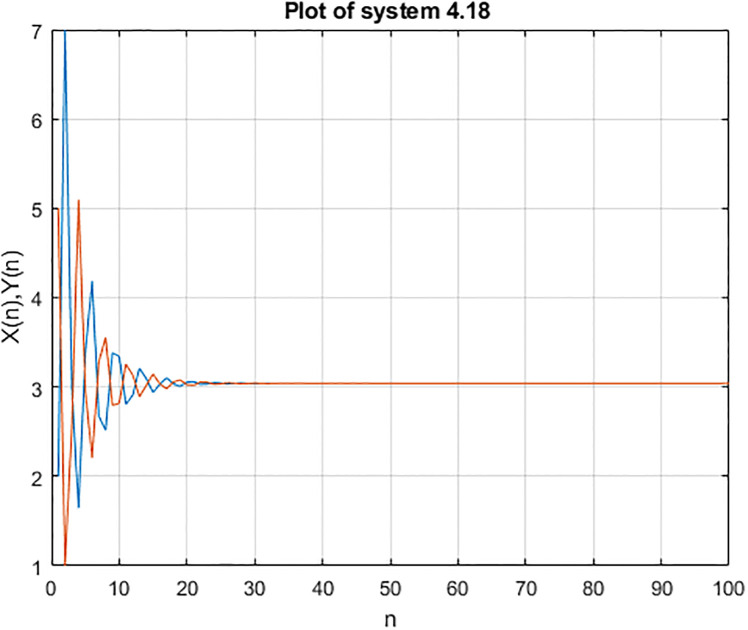
Solution of 78.

**Fig 8:**
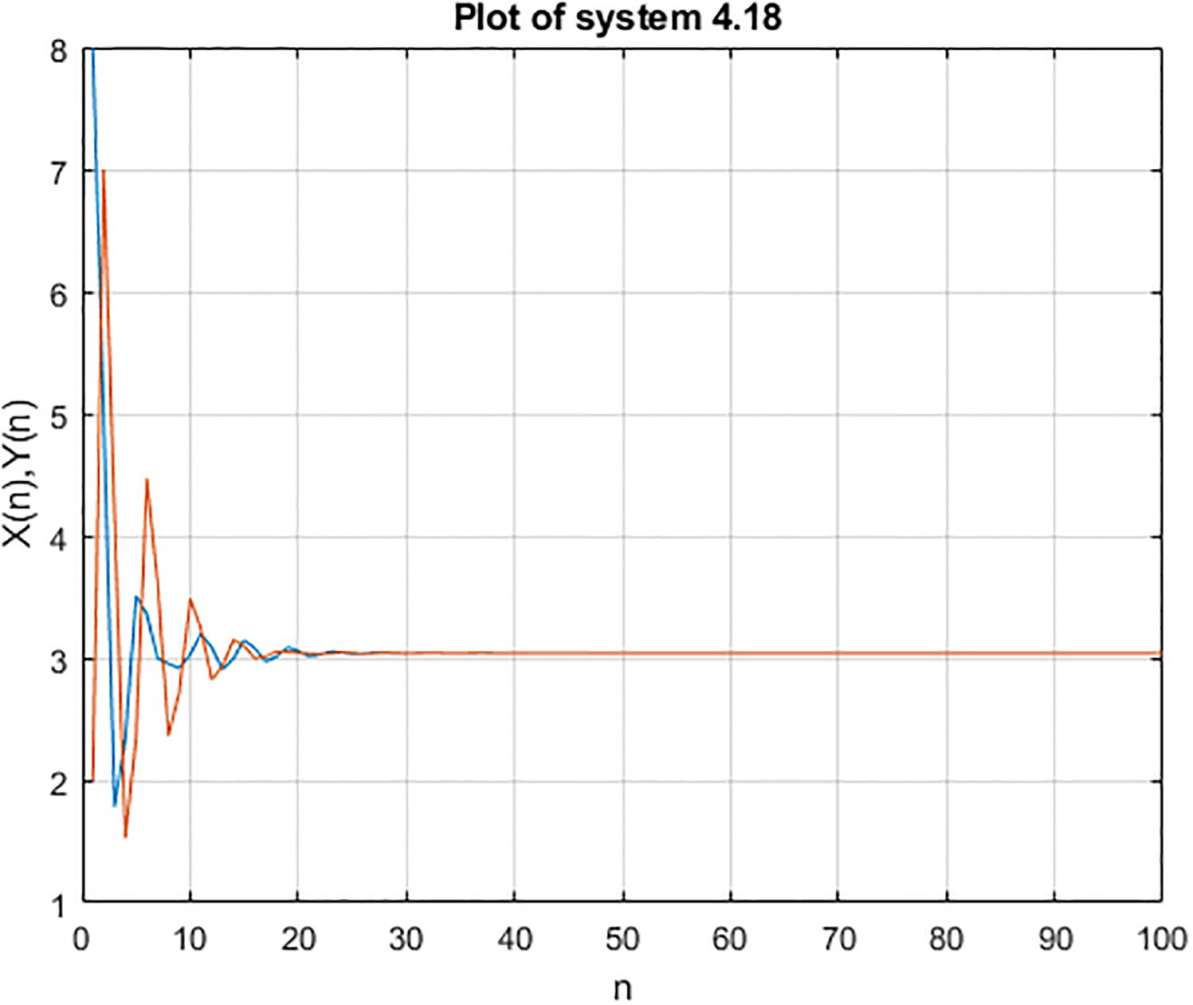
Solution of 78 b.

**Fig 9:**
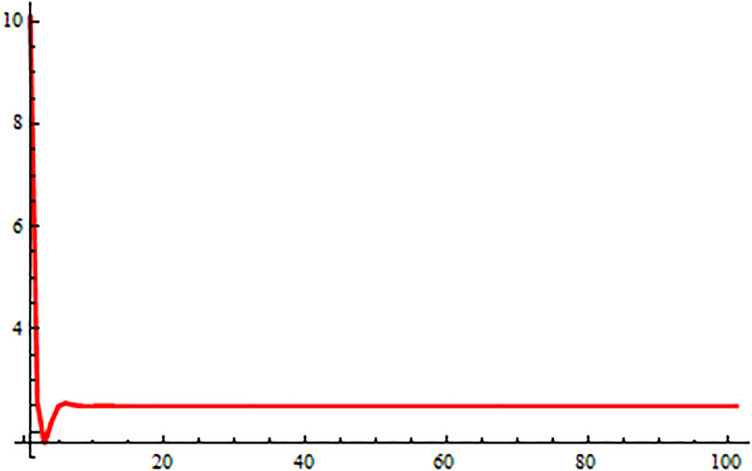
Solution of 80.

**Fig 10:**
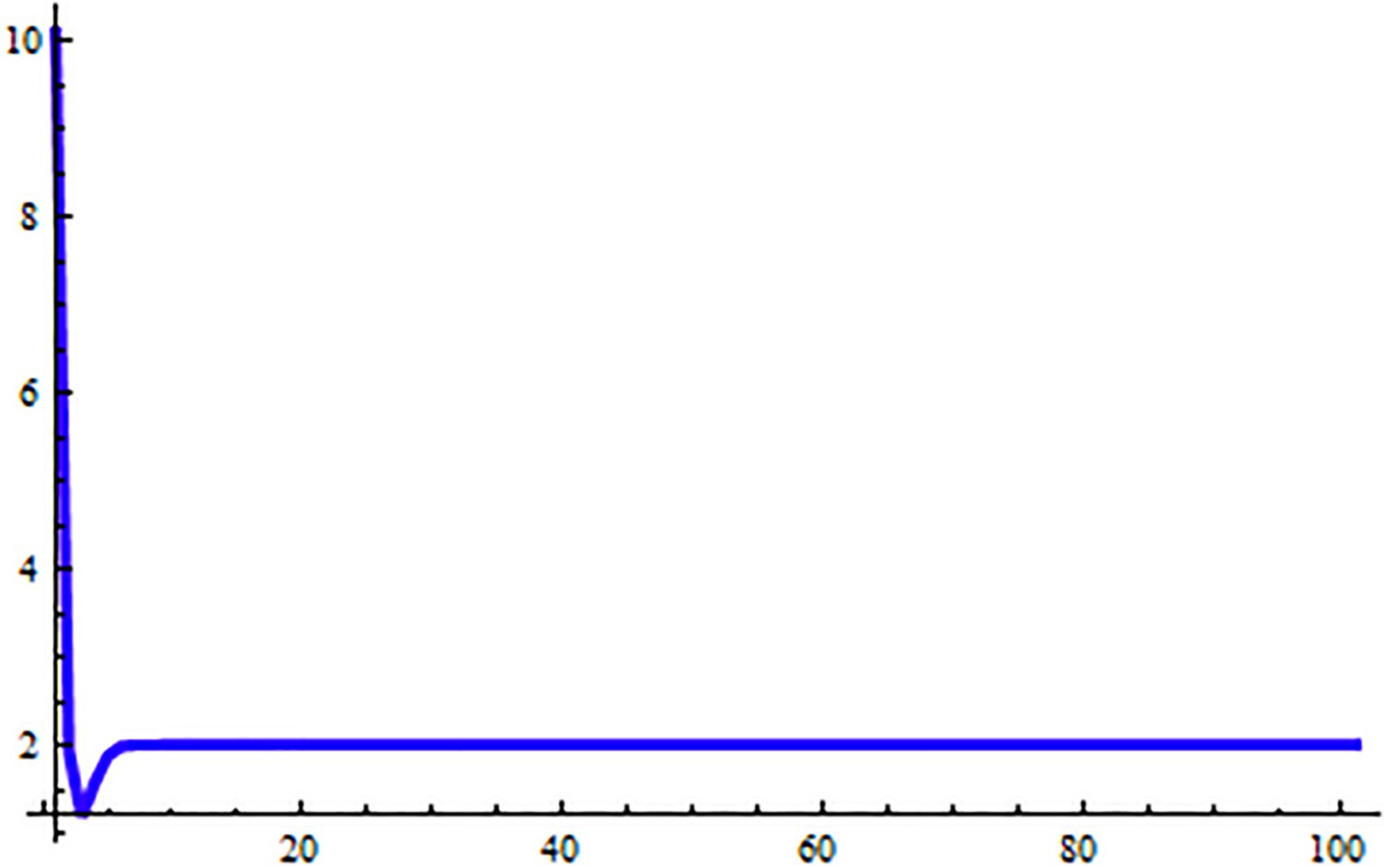
Solution of 80 b.

**Fig 11:**
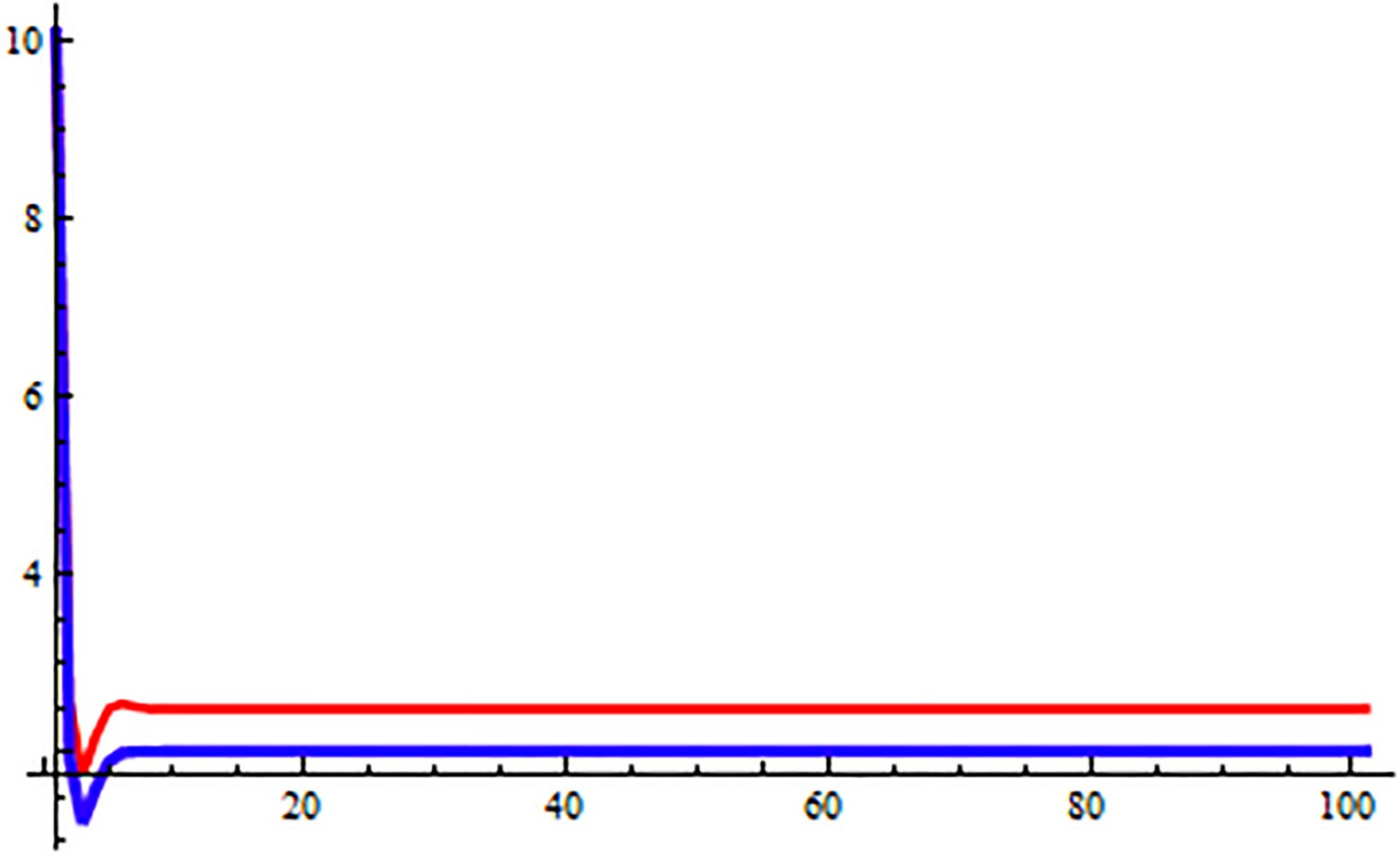
Solution behavior of 80.

**Fig 12:**
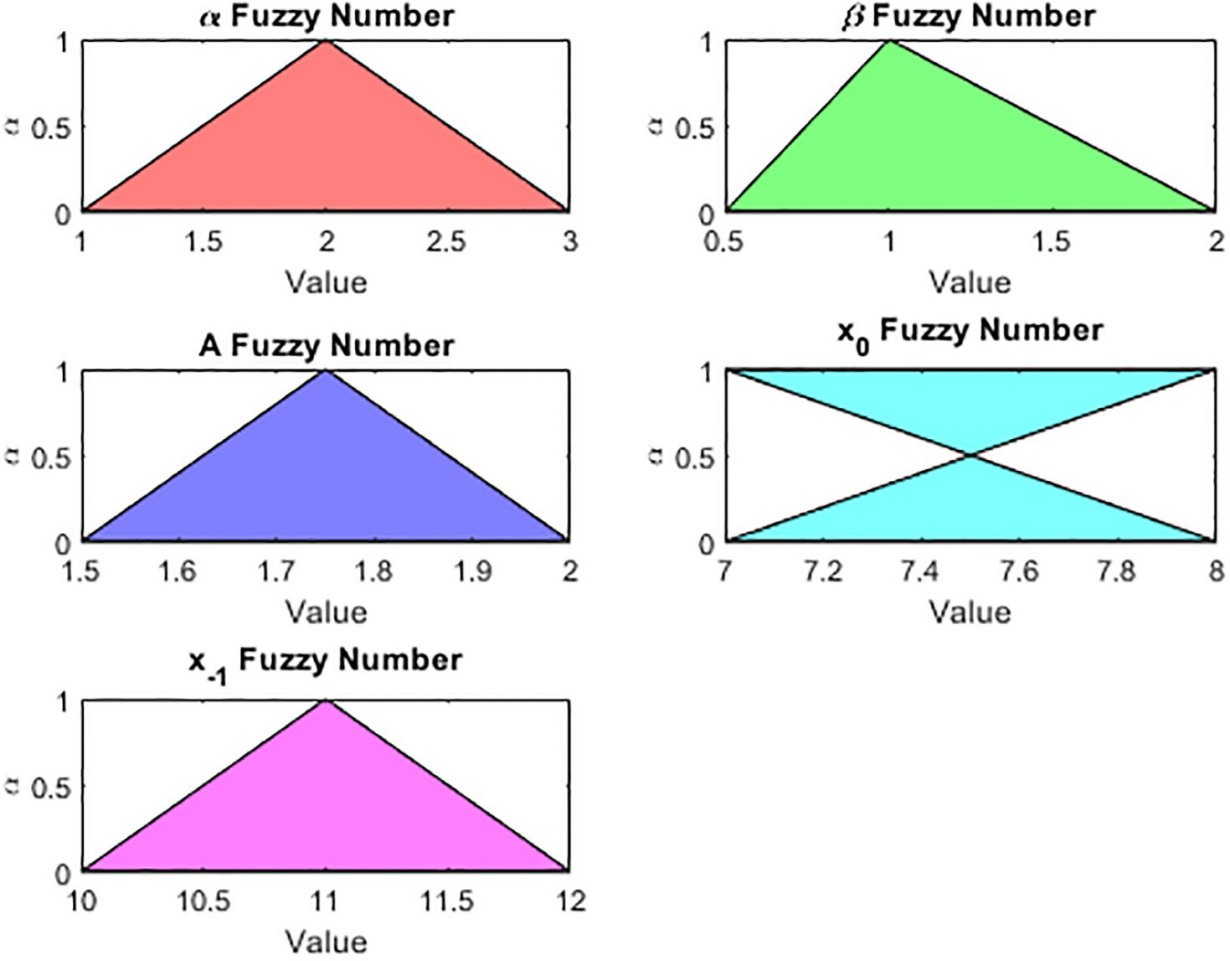
Solution behavior fo fuzzy numbers 82.

In the Figs 7–12, it shows that all required condition of theorems 57 holds true, so every +ve solution *x*_*n*_ of Eq 80 is exists and bounded, also from theorems 57, and Eq 80 has a unique +ve (EP) $\bar{x}=\left(0.0272,0.0272,0.0272\right)$. Moreover every +ve results *x*_*n*_ of 80 tends to $\bar{x}$ when *i* → ∞ see Figs 9–11. From Fig 11 we see that the +ve results *x*_*n*_, [*x*]_*α*_ = [*L*_*n*, *α*_, *R*_*n*, *α*_], of Eq 80 converge to a +ve (EP) $\bar{x}=\left(0.0272,0.0272,0.0272\right)$ as *n* → ∞.

**Example 4.5**.

$$\begin{array}{c} x_{n+1}=\frac{\alpha +\beta logx_{n}}{A+x_{n-1}},\;n\in W \end{array}$$ (81)

*Where α*, *β*, *A*, *x*_−1_, *and x*_0_ *be the fuzzy number then*

$$\begin{array}{c} {\left[\alpha \right]}_{\alpha }=\left[1+\alpha ,3-\alpha \right],\;{\left[\beta \right]}_{\alpha }=\left[0.5+0.5\alpha ,2-\alpha \right] \end{array}$$

$$\begin{array}{c} {\left[A\left(x\right)\right]}_{\alpha }=\left[1.5+0.25\alpha ,2-0.25\alpha \right],\;{\left[x_{0}\right]}_{\alpha }=\left[7+\alpha ,8-\alpha \right] \end{array}$$

$$\begin{array}{c} {\left[x_{-1}\right]}_{\alpha }=\left[10+\alpha ,12-\alpha \right] \end{array}$$

*and*

$$\begin{array}{c} \bar{\cup_{\alpha \in (0,1]}{\left[\beta \right]}_{\alpha }}=\left[0.5,2\right],\;\bar{\cup_{\alpha \in (0,1]}{\left[\alpha \right]}_{\alpha }}=\left[1,3\right] \end{array}$$

$$\begin{array}{c} \bar{\cup_{\alpha \in (0,1]}{\left[A\left(x\right)\right]}_{\alpha }}=\left[0.5,2\right],\;\bar{\cup_{\alpha \in (0,1]}{\left[x_{0}\right]}_{\alpha }}=\left[7,9\right] \end{array}$$

$$\begin{array}{c} \bar{\cup_{\alpha \in (0,1]}{\left[x_{-1}\right]}_{\alpha }}=\left[10,12\right] \end{array}$$

*Now by consider the DE from* 81 *when α cut is apply then we consider a couple of DE with parameter α which are given by*:

$$\begin{array}{cc} L_{n+1,\alpha } & =\frac{\alpha_{l,\alpha }+\beta_{l,\alpha }\times logR_{n,\alpha }}{A_{l,\alpha }+L_{n-1,\alpha }},\;R_{n+1,\alpha }\frac{\alpha_{r,\alpha }+\beta_{r,\alpha }\times logL_{n,\alpha }}{A_{r,\alpha }+R_{n-1,\alpha }},\;0<\alpha \leq 1 \end{array}$$ (82)

## 5 Conclusion and discussion

Engineering, ecology, social science, and other fields rely heavily on mathematical modeling to solve many real-life problems. When these real-life problems obey the continuity rule, they can be represented as differential equations, while difference equations are used to describe discrete systems. The use of difference equations in discrete system modeling has become increasingly important in the last few years. Meanwhile, uncertainty and imprecision are intrinsic to the problems that are faced in daily life, so fuzzy theory comes into play naturally in the boundary of difference equations. The study of (FDE) has already produced several interesting results, and more are on their way. In this article, we discussed how to solve a non-homogeneous linear (FDE) associated with fuzzy initial conditions, forcing functions, and fuzzy coefficients. Additionally, an equilibrium point and stability analysis of a model system presented by a fuzzy difference equation are discussed. In summary, the article contributes to and achieves the following:

A logarithmic type (FDE) of order two is investigated by using the g-division technique $x_{n+1}=\frac{\alpha +\beta logx_{n}}{A+x_{n-1}}$ Eq 1 is studied qualitatively and for its existence of positive solutions.

(*i*) In case I; There exists a bounded and +ve fuzzy solution to Eq 1. Additionally, if 26, and 27, are true, then every +ve solution of *x*_*n*_ leads to a unique (EP) $\bar{x}$ as *n* → ∞.

(*ii*) In case II; There exists a bounded and +ve fuzzy solution to Eq 1.

Further-more, if 40, 41, are true, then every +ve solution of *x*_*n*_ leads to a unique (EP) $\bar{x}$ as *n* → ∞.

The theoretical results are validated by five numerical examples. Several real-life situations require the use of second-order logarithmic fuzzy difference equations for predicting the future, such as population growth, stock prices, disease spread, product quality, climate change, and traffic flow. As a result of this equation, we can understand and forecast these changes by accounting for uncertainty and complexity, such as unexpected events or unclear measurements. While this could be advantageous, there are limitations, such as a requirement for large amounts of data and a sensitivity to parameter settings. As research advances, more efficient solutions should be developed, applications should be explored in new fields such as renewable energy and cybersecurity, and hybrid models should be studied to improve prediction accuracy. It is possible to improve the predictability and understanding of complex systems by addressing these limitations and expanding their applications.

## Abbreviations

DE: Difference Equation
EP: Equilibrium Points
FDE: Fuzzy Difference Equation
FN: Fuzzy Number
ODE: Ordinary Differential Equation
TNF: Triangular Fuzzy Number
